# *Chlamydomonas reinhardtii* exhibits stress memory in the accumulation of triacylglycerols induced by nitrogen deprivation

**DOI:** 10.1002/pei3.10069

**Published:** 2022-03-01

**Authors:** Pawel Mikulski, Javier Santos‐Aberturas

**Affiliations:** ^1^ Cell and Developmental Biology, John Innes Centre Norwich UK; ^2^ Molecular Microbiology, John Innes Centre Norwich UK

**Keywords:** algae, biofuel, *Chlamydomonas*, epigenetics, stress memory, triacylglycerols

## Abstract

Stress memory is a phenomenon whereby exposure to initial stress event influences a response to subsequent stress exposures. Studying stress memory is important to understand the cellular behavior in dynamic environment, especially nowadays, in times with growing environmental instability. Stress memory has been characterized in vascular plants but its occurrence in nonvascular plant species has been rarely investigated. We hypothesized that stress memory occurs in nonvascular plants in relation to metabolic stress. We sought to test it using accumulation of lipids (triacylglycerols) in model green alga *Chlamydomonas reinhardtii* subjected to nitrogen deprivation stress as a model system. Here, we established stress memory protocol on *C. reinhardtii* cells. Using a blend of microscopy and gas chromatography methods, we showed that the cells exposed to recurrent stress show differential accumulation of triacylglycerols on the quantitative level without qualitative changes in lipid composition, comparing to single stress controls. Overall, our results suggest that metabolic stress memory does occur in nonvascular plant *C. reinhardtii* and provides a starting point to characterize mechanistic principles of metabolic stress memory. Due to the commercial potential of algae, our findings are relevant for basic science, as well as industrial production of algae‐derived compounds.

## 1 INTRODUCTION

Stress memory is a phenomenon whereby exposure to initial stress event influences a response to subsequent stress exposures. Its importance is highlighted especially nowadays, in times with growing environmental instability (IPCC, 2018). Stress memory has been characterized in vascular plants (Mozgova et al., 2019), where it is related to, that is, response to drought (Ding et al., 2012) or temperature (Brzezinka et al., 2016). However, the occurrence of stress memory has been only rarely reported in nonvascular plants (Korkaric et al., 2015; Widiez et al., 2014), and still remains a largely unexplored niche.

Unicellular algae form a powerful biotechnological warehouse for production of chemical compounds. Triacylglycerols (TAGs) are lipids that can be used as the precursors in biodiesel production for academic and industrial research. Model green alga, *Chlamydomonas reinhardtii*, accumulates TAGs in stress conditions such as nitrogen deprivation (Park et al., 2015). TAGs accumulation is reversible (Roustan et al., 2017) and chromatin‐based (Ngan et al., 2015), features that make this process potentially able to demonstrate stress memory.

How TAGs' accumulation behaves in *C. reinhardtii* subjected to multiple nitrogen deprivation events and whether the species exhibits metabolic stress memory has not been investigated. We hypothesized that stress memory occurs in the accumulation of TAGs in *C. reinhardtii* subjected to nitrogen deprivation. Here, we established a stress memory growth protocol in *C. reinhardtii*. Using a blend of microscopy and gas chromatography, we demonstrated that algal cells exposed to recurrent stress show attenuated stress‐induced accumulation of triacylglycerols on the quantitative level, comparing to single stress controls. However, this differential accumulation does not involve qualitative changes in lipid composition. Our results suggest that model green algae cells exhibit phenotypic memory and potential acclimation to recurrent nitrogen deprivation stress.

This work provides a proof‐of‐principle evidence that metabolic stress memory does occur in nonvascular plant *C. reinhardtii* and opens a new avenue to understand the mechanisms behind stress memory and cellular response to the fluctuating environment. Due to commercial potential of algae and usage of stress‐induced processes for production of biologics, this work could influence academic and industrial researchers.

## 2 MATERIAL AND METHODS

### 2.1 Growth conditions and growth medium

Wildtype *C. reinhardtii* strain 137c was grown in liquid cultures on TAP medium with agitation at 180 rpm as described previously (Ngan et al., 2015). For nitrogen depletion, TAP medium without nitrogen source was used as described in (Ngan et al., 2015). The cells were washed twice with nitrogen‐depleted medium before media change during stress treatment. The cells were passaged twice and inoculated in a fresh medium between stress induction timepoints. Alga cultures were kept in growth cabinets (Sanyo‐MLR‐352‐PE) set for 25°C and supplied with LED fluorescent lights (Newlec, NL/18/LED/T8/4/865 & 840).

### 2.2 Fixation and Nile red staining

We performed TAGs' staining with Nile red dye on fixed cells. We used 3% (v/v) formaldehyde on 1 ml culture aliquots, incubated for 20 min on ice, spun at 1000 *g* for 5 min and washed twice with phosphate saline buffer (PBS). Final cell pellet was resuspended in 20–50 μl PBS based on estimation from OD600. Final solution of 6–7 μl was spread onto microscopy slides, air dried and refrigerated until staining. Staining was done with 5 μg/ml in 0.1% acetone for 15 min in dark.

### 2.3 Microscopy and image analysis

Image acquisition was performed in two channels: Ex488/Em520‐600 and Ex633/Em635‐700 (Ex = excitation, Em = emission), where former corresponds to specific Nile red signal and the latter to cell autofluorescence. For intensity quantifications, Ex488/Em520‐600 channel was used to avoid a crosstalk with autofluorescence channel. For Z‐stacks, we used a step size (*z*‐distance between slices) of 0.55–0.59 μm and line averaging at 2. Following parameters for image acquisition were used: scan speed at 400 Hz, pinhole size at 1 Airy unit and laser power at 16%. Image analysis was performed in Fiji/ImageJ. Nile red intensity quantification was done in three biological replicates per harvesting point and sample type. We quantified intensity from 276 to 665 cells per condition: ZT2 N−−, ZT4 N+−, or ZT4 N−−. Statistical analysis was done using Student's *T*‐test with the calculation of the variance over multiple biological replicates within respective condition.

### 2.4 Total lipid extraction

From 100 ml culture in each time point (ZT), we harvested 2 ml aliquot and centrifuged it (1500 *g*, 10 min) in 20 ml glass vials. The supernatant was discarded and cell pellet equalized between the samples. The pellet was mixed with 1 ml of chloroform/methanol 2:1 by vigorous vortexing (2500 rpm, 30 min) and then centrifuged (1900 *g*, 10 min). Resuspended pellet of 500 μL was transferred to clean 2 ml glass vial, vortexed (2500 rpm, 20 min) with 200 μl of mass‐spectrometry‐grade water to extract polar molecules and left for gravity phase separation. The lower, organic layer containing lipids was transferred to clean 2 ml gas chromatography mass spectrometry (GC–MS) glass vial and evaporated to dryness in a GeneVac evaporator, being finally ready for analysis by GC–MS.

### 2.5 Gas chromatography mass spectrometry

GC–MS analyses were performed on an Agilent 7890B GC system coupled to an Agilent 5977A Mass Selective Detector, using a Phenomenex Zebron ZB5‐HT Inferno (35 m × 250 μm × 0.1 μm). The TAGs and other fatty acid esters contained in the dry samples were resuspended and derivatized with methanol into fatty acid methyl esters via alkali catalysis in an automated fashion before the injection of each sample. The injector and interface were operated at 275 and 230°C, respectively, and the oven heated from 50 to 260°C at a rate of 7°C min^−1^ and then isothermally held for 5 min. The total run time for each sample was 37 min. Helium was used as carrier gas, with a flow of 2 ml min^−1^. Sample of 1 μl was injected in splitless mode. The MS was operated in scan mode (50–500 Da), with an ionization energy of 70 eV.

## 3 RESULTS

### 3.1 Establishment of stress memory growth setup

As a first step, we sought to establish stress memory growth protocol for *C. reinhardtii*. We optimized light intensity conditions (Figure S1a) and tested culture growth in medium depleted of nitrogen source or in default TAP medium with standard nitrogen source concentration (Ngan et al., 2015). As expected, our test showed substantial doubling and increased OD600 for cultures grown in default medium, but not in the nitrogen‐depleted samples (Figure S1b).

**FIGURE 1:**
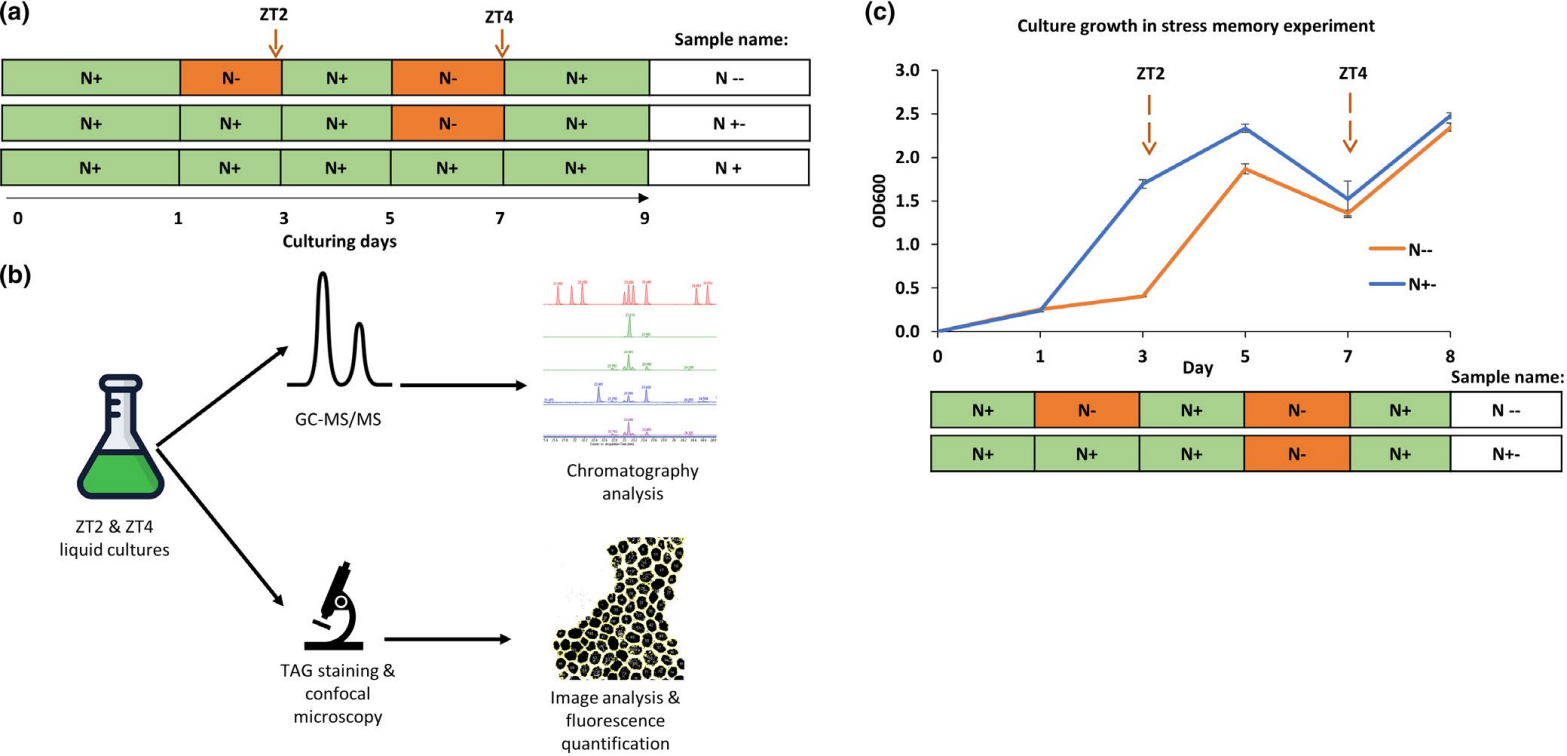
Stress memory growth schematic and cell growth. (a). Stress memory growth scheme. ZT (*zeitgaber*) indicate harvesting timepoints for triacylglycerols (TAG) measurements. N+ and N‐ labels correspond to nitrogen‐control and nitrogen‐depleted growth conditions, respectively. (b). Postharvesting workflow schematic **c.** growth measurements (OD600) of cells in stress memory experiment. Error bars correspond to standard error from three biological replicates (separate flasks)

Data published previously suggest that lipid accumulation in stressed *C. reinhardtii* cells reaches substantial levels already after 2 day‐long growth under nitrogen depletion without lethal effect in the culture (Ngan et al., 2015). Therefore, we designed stress priming setup, allocating 2 days for stress treatment and 2 days for the recovery period (Figure 1a). We set samples to be treated with two nitrogen depletion events (stress primed) or single stress event (control), harvesting aliquots for further analyses at timepoints (*zeitgeber*): ZT2 and ZT4, as indicated on the scheme (Figure 1a). We used three biological replicates for each timepoint in nitrogen‐deprived and nitrogen‐control samples and harvested aliquots for microscopy or GC–MS/MS (Figure 1b). To exclude the possibility that 1st nitrogen depletion event interferes with a readout from 2nd nitrogen depletion and to ensure constant cell growth, we washed cultures with TAP‐N (nitrogen‐depleted medium) before each stress event and split the cells 1:1 directly after 1st stress (after ZT2) and at the end of the recovery window before 2nd stress (before ZT4). We observed a decrease in OD600 after ZT4, but it was present in both, nitrogen‐depleted and the control samples, probably coming from the stress event itself and the cell dilution due to the passaging at the end of the memory phase. Importantly, the cells recover well and continue proliferation after ZT4 (day 8, Figure 1c). Our OD600 measurements suggested that the cells were actively dividing during stress memory growth setup (Figure 1c). The cells temporally ceased proliferation only during stress events and recovered after nitrogen repletion, indicating that they were not exhausted after initial nitrogen depletion.

### 3.2 Optimization of TAG staining and image analysis workflow

**FIGURE 2:**
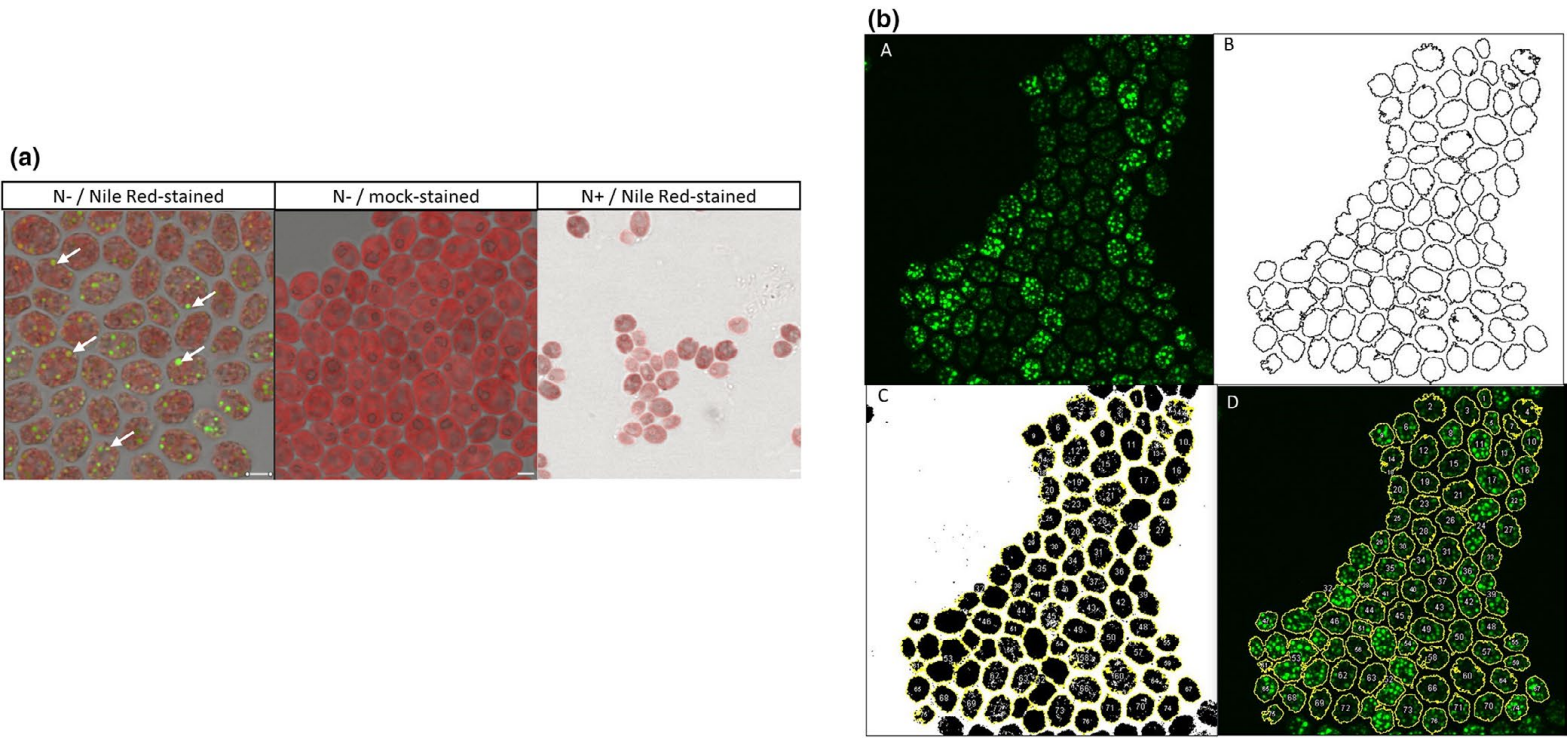
Triacylglycerol (TAG) staining and microscopy workflow. (a). Representative images for Nile red‐ and mock‐stained samples. N− label corresponds to nitrogen‐depleted samples, whereas N+ to control with standard nitrogen source concentration. The arrows correspond to examples of lipid bodies specifically stained by Nile red. (b). Image analysis workflow: (a) Z‐projection for Nile red‐specific channel; (b) cell segmentation with pixel intensity thresholding; (c) cell annotation on binary image (d) cell annotation on original image for intensity quantification. Scale bar corresponds to 5 μm

Using previously harvested aliquots at indicated timepoints, we sought to measure TAGs accumulation through histochemistry, microscopy, and image analysis. First, we performed chemical fixation of cells and their Nile Red staining of TAGs (see Section 2). Nile Red is an efficient TAG marker and its fluorescence was reported to be tightly correlated with TAG concentration in *C. reinhardtii* (Johnson et al., 2017). Afterwards, we optimized confocal microscopy conditions (see Section 2) to separate Nile Red signal from cellular autofluorescence and acquired Z‐stacks of prepared specimens to capture Nile Red signal in 3D. We observed only background autofluorescence in negative microscopy controls (mock‐stained nitrogen‐depleted (N−) or Nile Red‐stained nitrogen‐control (N+) samples) without specific Nile Red signal (Figure 2a). In contrast, Nile Red‐stained nitrogen‐depleted specimen showed TAGs' accumulation in lipid bodies, as reported previously (Wang et al., 2009), and selective Nile Red signal (Figure 2a), which confirmed specificity of staining and microscopy workflow.

To properly quantify Nile Red‐stained TAG accumulation, we established a workflow for image analysis. First, we converted Z‐stack to single images by slice summing in Z‐projection. Afterwards, we segmented cells with minimum error threshold to create masks used as regions‐of‐interest (ROIs) outlining cell borders. Finally, we quantified mean fluorescence intensity in gray value inside ROIs as a way to measure Nile Red‐stained TAG intensity. The examples of different workflow stages are present in Figure 2b.

### 3.3 TAG intensity quantification

Following measurement of a raw Nile Red‐stained TAGs' fluorescence intensity, we normalized technical differences between the images. To this end, we quantified background fluorescence outside the cells on the image and subtracted its value from the specific Nile Red signal from within the cells. We used normalized intensity measurements compare stress‐primed sample at ZT4 (second stress exposure) with its controls: stress‐memory samples at ZT2 (first stress exposure, ZT2 N−−) and single‐stressed samples at ZT4 (ZT4 N+−). To ensure statistical power of the analysis, each sample had three biological replicates and the intensity measurements in each biological replicate was done on 276–665 cells per condition.

**FIGURE 3:**
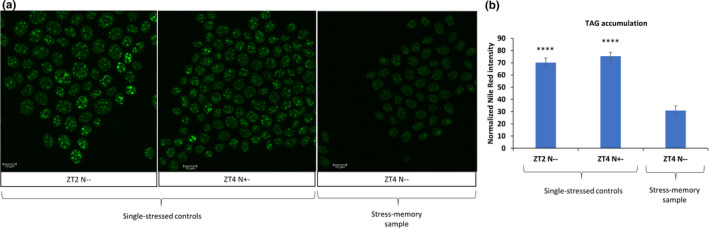
Imaging of triacylglycerols (TAGs) accumulation (a) Representative images of TAG‐stained cells from stress‐memory samples (N−−) and single‐stressed controls (N+− and N−− at first nitrogen depletion). Scale bar corresponds to 10 μm. (b) Nile red intensity quantification as a proxy for TAG accumulation measurements. Error bars correspond to standard error from three biological replicates and statistics were calculated using Student's *t*‐test in comparison: ZT2 N−− to ZT4 N−− and ZT4 N+− to ZT4 N−−

As a result, we observed a clear and significant difference between stress‐primed samples and single‐stressed controls. Stress‐primed samples showed downregulation of TAGs' fluorescence intensity (Figure 3a,b), suggesting a differential response to recurrent nitrogen deprivation conditions and a memory effect of previously encountered stress. We do not envisage that an observed effect at ZT4 (second stress) is a technical bleed‐through from ZT2 (first stress), nor an effect of exhaustion, since cells has been passaged twice before ZT4 and inoculated in fresh cultures (methods). Importantly, the cultures were actively dividing throughout the experiment and did not cease proliferation between stress events (Figure 1c). In turn, this observation in such experimental setup suggests a memory effect that is heritable over cell division and potentially of epigenetic nature.

### 3.4 TAG profiling using GC–MS/MS

To qualitatively assess the lipid composition of the accumulated TAGs and complement the confocal microscopy measurements, we performed a GC–MS lipidomic analysis. We extracted lipids from stress‐primed cultures (ZT4 N−/−) and single‐stressed control harvested at the same time (ZT4 N+/−). The extracted lipids underwent transesterification to generate volatile fatty acid methyl esters (see methods), which were subsequently analyzed by GC–MS, along with standard fatty acid libraries.

**FIGURE 4:**
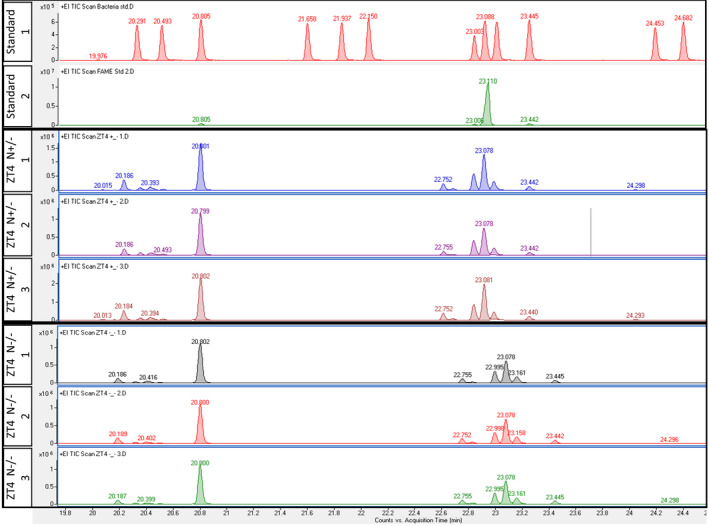
Triacylglycerol (TAG) profiling through gas chromatography mass spectrometry (GC–MS)/MS. GC–MS TAG profiling. Chromatograms depict retention peaks of single‐stressed ZT4 N+− and stress‐primed ZT4 N−− samples, each in three biological replicates as indicated in the numbers on the side labels. Two top bars correspond to GC–MS/MS lipid standards

Our results reveal that the qualitative profile of lipid types did not show substantial differences between stress‐primed samples and single‐stressed controls (Figure 4), despite different lipid accumulation observed through histochemistry. This observation suggests that quantitative stress priming response in TAGs accumulation does not come with qualitative change in lipid composition.

## 4 DISCUSSION

In summary, we observed a differential TAG accumulation caused by stress memory in *Chlamydomonas* cells. Cells subjected to two rounds of nitrogen depletion events show attenuated TAG accumulation, compared to single‐stressed counterparts. Interestingly, this change was observed on quantitative level, as judged from histochemical measurements, but not in qualitative fashion––stress‐memory samples do not exhibit substantial differences in the abundance of lipid subtypes, comparing to the controls. TAGs quantitative downregulation in stress‐memory samples indicates that cells exhibit attenuated nitrogen deprivation response based on the previous stress exposure or respond to further stress events with pathways antagonistic to TAGs' accumulation. Given the ongoing growth of the culture throughout the experiment, we envisage that observed response in stress‐memory samples is potentially of epigenetic nature. Future steps should aim to elucidate the mechanism of stress memory and identify its causal factors.

In conclusion, our results highlight that metabolic stress memory of nitrogen depletion exists in model green alga and suggests that metabolic stress memory mechanisms could be widely conserved in nonvascular plants.

## CONFLICT OF INTEREST

The authors declare no conflict of interest.

## Supporting information


DataS1

FigS1

## Data Availability

The data that supports the findings of this study are available in the supplementary material of this article and on Mendley Data under: doi: 10.17632/98gnch8zp8.1.
