# Supplementary material for: Chlamydomonas reinhardtii exhibits stress memory in the accumulation of triacylglycerols induced by nitrogen deprivation
Source: Plant Environ Interact. 2022 Mar 1;3(1):10–5. doi: 10.1002/pei3.10069 (PMC10168029; doi:10.1002/pei3.10069)

**Supporting information**

Supporting Information to the paper Matinzadeh, Z. Functional structure of plant communities along salinity gradients in Iranian salt marshes. *Plant-Environment Interactions*.

Appendix S1. Details of studied plots, their location, geodesic coordinates, altitude, salt marsh names and dominance plant species.

| **Plots** | **Address** | **Geodesic coordinates** | **Altitude (m)** | **Salt marsh name** | **Dominant plant species** |
| --- | --- | --- | --- | --- | --- |
| **U11** | East Azarbaijan; Azarshahr | 37°42'32.2"N, 45°50'24.4"E | 1301 | Lake Urmia | *Halocnemum strobilaceum* & *Halimocnemis rarifolium* |
| **U12** | East Azarbaijan; Azarshahr | 37°42'29.1"N, 45°50'27.1"E | 1298 | Lake Urmia | *Aeluropus littorali*s & *Halimioine verrucifera* |
| **U13** | East Azarbaijan; Azarshahr | 37°42'30.7"N, 45°50'29.1"E | 1301 | Lake Urmia | *Juncus heldreichianus* |
| **U14** | East Azarbaijan; Azarshahr | 37°42'30"N, 45°50'29.3"E | 1309 | Lake Urmia | *Juncus heldreichianus* |
| **U15** | East Azarbaijan; Azarshahr | 37°42'29.1"N, 45°50'30.3"E | 1303 | Lake Urmia | *Juncus heldreichianus* |
| **U16** | East Azarbaijan; Azarshahr | 37°42'29.4"N, 45°50'34.1"E | 1300 | Lake Urmia | *Juncus heldreichianus* |
| **U17** | East Azarbaijan; Azarshahr | 37°42'24"N, 45°50'43"E | 1284 | Lake Urmia | *Lycium ruthenicum* |
| **U18** | East Azarbaijan; Azarshahr | 37°42'22"N, 45°50'37"E | 1297 | Lake Urmia | *Halimioine verrucifera* |
| **U19** | East Azarbaijan; Azarshahr | 37°42'21.8"N, 45°50'35.3"E | 1331 | Lake Urmia | *Alhagi maurorum* |
| **U110** | East Azarbaijan; Azarshahr | 37°42'22"N, 45°50'38.3"E | 1302 | Lake Urmia | *Caroxylon dendroides* |
| **U111** | East Azarbaijan; Azarshahr | 37°42'16.4"N, 45°50'47"E | 1314 | Lake Urmia | *Artemisia spicigera* |
| **U21** | East Azarbaijan; Islami Island | 37°49'49.3"N, 45°35'13.7"E | 1291 | Lake Urmia | *Climacoptera crassa* |
| **U22** | East Azarbaijan; Islami Island | 37°49'51.3"N, 45°35'10.0"E | 1290 | Lake Urmia | *Suaeda altissima* |
| **U23** | East Azarbaijan; Islami Island | 37°49'51.5"N, 45°35'07.2"E | 1292 | Lake Urmia | *Suaeda altissima* |
| **U24** | East Azarbaijan; Islami Island | 37°49'47.9"N, 45°35'02.9"E | 1292 | Lake Urmia | *Alhagi maurorum* |
| **U31** | East Azarbaijan; Akhondgheshlagh | 37°22'03.2"N, 45°59'06.2"E | 1291 | Lake Urmia | *Climacoptera crassa* |
| **U32** | East Azarbaijan; Akhondgheshlagh | 37°21'52.5"N, 45°59'31.6"E | 1290 | Lake Urmia | *Climacoptera crassa* & *Atriplex tatarica* |
| **U33** | East Azarbaijan; Akhondgheshlagh | 37°21'50.8"N, 45°59'36.4"E | 1281 | Lake Urmia | *Climacoptera crassa* |
| **U34** | East Azarbaijan; Akhondgheshlagh | 37°21'48.2"N, 45°59'41.7"E | 1290 | Lake Urmia | *Alhagi maurorum* |
| **U35** | East Azarbaijan; Akhondgheshlagh | 37°21'42.7"N, 46°00'03"E | 1292 | Lake Urmia | *Halocnemum strabilaceum* |
| **U41** | West Azarbaijan; Rashakan | 37°08'38.7"N, 45°26'19.5"E | 1284 | Lake Urmia | *Salicornia iranica* |
| **U42** | West Azarbaijan; Rashakan | 37°08'37.3"N, 45°26'16.1"E | 1272 | Lake Urmia | *Juncus heldreichianus* & *Typha grossheimii* |
| **U43** | West Azarbaijan; Rashakan | 37°08'35.4"N, 45°26'14.8"E | 1291 | Lake Urmia | *Alhagi maurorum* & *Cynanchum acutum* |
| **U51** | East Azarbaijan; Gol Tappeh | 37°54'04.0"N, 45°03'31.0"E | 1281 | Lake Urmia | *Climacoptera crassa* |
| **U52** | East Azarbaijan; Gol Tappeh | 37°54'003"N, 45°03'15.7"E | 1278 | Lake Urmia | *Ceratocephalus falcatus* & *Lepidium vesicarium* |
| **U53** | East Azarbaijan; Gol Tappeh | 37°53'58.3"N, 45°03'07.4"E | 1281 | Lake Urmia | *Zygophyllum fabago* |
| **U54** | East Azarbaijan; Gol Tappeh | 37°53'56.8"N, 45°03'02.2"E | 1285 | Lake Urmia | *Descurainia sophia* |
| **U55** | East Azarbaijan; Gol Tappeh | 37°53'54.0"N, 45°02'51.1"E | 1277 | Lake Urmia | *Suaeda altissima* |
| **U56** | East Azarbaijan; Gol Tappeh | 37°53'53.8"N, 45°02'47.4"E | 1275 | Lake Urmia | *Tamarix octandra* |
| **U6** | West Azarbaijan; Chi chest | 37°34'39.5"N, 45°15'49.6"E | 1283 | Lake Urmia | *Halimocnemis rarifolium* & *Aeluropus littoralis* |
| **U7** | East Azarbaijan; Saray | 37°51'48.6"N, 45°34'45.0"E | 1285 | Lake Urmia | *Soda inermis* |
| **U81** | West Azarbaijan; Soldouz | 37°02'08.4"N, 45°35'15.3"E | 1292 | Lake Urmia | *Salicornia iranica* |
| **U82** | West Azarbaijan; Soldouz | 37°02'08.5"N, 45°35'16.3"E | 1285 | Lake Urmia | *Bolboschoenus affinis* |
| **U83** | West Azarbaijan; Soldouz | 37°02'09.6"N, 45°35'16.9"E | 1288 | Lake Urmia | *Phragmites australis* |
| **N11** | Bushehr; Nayband | 27°26'41.1"N, 52°40'32.3"E | 2.5 | Persian Gulf | *Halopyrum mucronatum* |
| **N12** | Bushehr; Nayband | 27°26'40.7"N, 52°40'33.9"E | 2.8 | Persian Gulf | *Heliotropium bacciferum* & *Senecio glaucus* |
| **N13** | Bushehr; Nayband | 27°26'44.2"N, 52°40'35"E | 11.3 | Persian Gulf | *Bolboschoenus glaucus* & *Hippocrepis bisiliqua* |
| **N14** | Bushehr; Nayband | 27°26'56.2"N, 52°40'31.6"E | 17.9 | Persian Gulf | *Avicennia marina* |
| **N15** | Bushehr; Nayband | 27°26'45.6"N, 52°40'36.3"E | 15.6 | Persian Gulf | *Limonium failachicum* & *Sporobolus arabicus* |
| **N16** | Bushehr; Nayband | 27°26'56.6"N, 52°40'33.8"E | 0.3 | Persian Gulf | *Arthrocaulon macrostachyum* |
| **N21** | Bushehr; Zobar | 27°22'22.1"N, 52°42'33.1"E | 10.7 | Persian Gulf | *Halimioine verrucifera* & *Halocharis sulfurea* |
| **N22** | Bushehr; Zobar | 27°22'20.30"N, 52°42'31.40"E | 12.8 | Persian Gulf | *Lycium shawii* |
| **N3** | Bushehr; Basatin | 27°23'29.3"N, 52°39'29.1"E | 10.2 | Persian Gulf | *Mesembryanthemum nodiflorum* & *Ziziphus nummularia* |
| **N4** | Bushehr; Chah Mobarak | 27°21'14.8"N, 52°46'09.9"E | 15.1 | Persian Gulf | *Halocnemum strobilaceum* & *Psylliostachys spicata* |
| **N5** | Bushehr; Banood | 27°21'52.3"N, 52°45'01.4"E | 10.6 | Persian Gulf | *Soda drummondii* |
| **K** | Khuzestan; Mousa estuary | 30°26'06, "N, 49°01'56.8"E | 1.5 | Persian Gulf | *Halocnemum strobilaceum* |
| **M1** | Ostane Markazi; Meyghan | 34°16'7"N, 49°45'17"E | 1692 | Lake Meyghan | *Nitraria schoberi & Atriplex canescens* |
| **M2** | Ostane Markazi; Meyghan | 34°10'14"N, 49°47'28"E | 1695 | Lake Meyghan | *Bienertia cycloptera* |

Supporting information to the paper Matinzadeh, Z. Functional structure of plant communities along salinity gradients in Iranian salt marshes. *Plant-Environment Interactions*.

Appendix S2. Details of plant species, abbreviation, study plots and categorical traits: life history, growth form, life form, photosynthetic pathways, salt tolerance categories and eco-morphotypes. Life history included Perennials (P), Annuals (A), Biennial (BA), Annual/Biennial (A/BA); growth form included Herbaceous (H), Shrub (SH), Sub-shrub (Sub-sh), Tree (T) and Woody-Climber (WC); life form included Chamaephyte (Ch), Hemi-cryptophyte (H), Geophyte (G), Therophyte (T), Helophyte (He), Phanerophyte (P); photosynthetic pathway included C_3_, C_4_ and C_3_-CAM; Salt-tolerance categories included Eu-halophytes (Eu), Facultative-halophytes (Fac), Eury-Hygro-halophytes (Eur), and Pseudo-halophytes (Pse); and the eco-morphotypes included Leaf-succulent (Leaf-suc), Stem-succulent (Stem-suc), Semi-succulent (Semi-suc), Salt-recreting (Salt-rec), and Non-succulent (Non-suc). The scientific name of those species changed after Flora Iranica (Rechinger 1963-2015) are marked with an asterisk (*) with relevant older synonym in the parenthesis.

|  | **Species Name** | **Abbr.** | **Plots** | **Life history** | **Growth form** | **Life form** | **Photosynth pathway** | **Salt-tolerant** | **Eco-Morphotype** |
| --- | --- | --- | --- | --- | --- | --- | --- | --- | --- |
| 1 | *Achillea tenuifolia* Lam. | Ach.ten | U110, U16, U18, U19 | P | H | H | C_3_ | Fac | Non-suc |
| 2 | *Acinos graveolence* Link | Aci.gra | U52 | A | H | Th | C_3_ | Pes | Non-suc |
| 3 | *Adonis annua* L. | Ado.ann | U19, U119, U16, U56 | A | H | Th | C_3_ | Pes | Non-suc |
| 4 | *Aeluropus lagopoides* (L.) Thwaites | Ael.lag | N22 | P | H | H | C_4_ | Eu | Salt-rec |
| 5 | *Aeluropus littoralis* (Gouan) Parl. | Ael.lit | U110, U12, U15, U16, U18, U19, U6 | P | H | H | C_4_ | Eu | Salt-rec |
| 6 | *Alhagi maurorum* Medik. | Alh.mau | U16, U17, U19, U24, U34, U43 | P | H | H | C_3_ | Fac | Non-suc |
| 7 | *Alopecurus myosuroides* Huds. | Alo.myo | U53 | A | H | Th | C_3_ | Eur | Non-suc |
| 8 | *Alyssum dasycarpum* Stephan ex Willd. | Aly.das | U52 | A | H | Th | C_3_ | Pes | Non-suc |
| 9 | *Alyssum desertrum* Stapf. | Aly.des | U18, U52 | A | H | Th | C_3_ | Pes | Non-suc |
| 10 | *Alyssum linifolium* Willd. | Aly.lin | M1, U16, U51 | A | H | Th | C_3_ | Fac | Non-suc |
| 11 | *Androsace maxima* L. | And.max | U16, U5 | A | H | Th | C_3_ | Pes | Non-suc |
| 12 | *Anthemis australis* Willd. | Ant.aus | U52 | A | H | Th | C_3_ | Pes | Non-suc |
| 13 | *Arenaria leptoclados* Guss. | Are.lep | U6 | A | H | Th | C_3_ | Pes | Non-suc |
| 14 | *Arnebia decumbens* (Vent.) Coss. & Kralik | Arn.dec | M1 | A | H | Th | C_3_ | Fac | Non-suc |
| 15 | *Artemisia sp.* | Art.sp. | U52 | P | Sub-Sh | Ch | C_3_ | Pes | Non-suc |
| 16 | *Artemisia sp.* | Art.sp. | M1 | P | Sub-Sh | Ch | C_3_ | Eu | Non-suc |
| 17 | *Artemisia spicigera* K.Koch | Art.spi | U110, U111 | P | Sub-Sh | Ch | C_3_ | Pes | Non-suc |
| 18 | *Arthrocaulon* *macrostachyum*(Moric.) Piirainen & G.Kadereit (=*Arthrocnemum macrostachyum* (Moric.) K. Koch)* | Art.mac | N16 | P | Sh | Ch | C_3_ | Eu | Stem-suc |
| 19 | *Asparagus verticillatus* L. | Asp.ver | U16 | P | HC | H | C_3_ | Fac | Non-suc |
| 20 | *Asperugo procumbens* L. | Asp.pro | U16, U52, U6 | A | H | Th | C_3_ | Pes | Non-suc |
| 21 | *Astragalus crenatus* Schult. | Ast.cre | N5, U110, U16, U19 | A | H | Th | C_3_ | Fac | Non-suc |
| 22 | *Atriplex canescens* (Pursh) Nutt. | Atr.can | M1 | P | Sh | Ch | C_4_ | Eu | Salt-rec |
| 23 | *Atriplex leucoclada* Boiss. | Atr.leu | M1, U110, U16, U17, U19 | P | Sub-Sh | Ch | C_4_ | Eu | Salt-rec |
| 24 | *Atriplex micrantha* C.A.Mey. | Atr.mic | U55 | A | H | Th | C_3_ | Eu | Salt-rec |
| 25 | *Atriplex tatarica* Auct. | Atr.tat | U31, U32, U33 | A | H | Th | C_4_ | Eu | Salt-rec |
| 26 | *Avena ludoviciana* Durieu | Ave.lud | N5 | A | H | Th | C_3_ | Pes | Non-suc |
| 27 | *Avicennia marina* (Forssk.) Vierh. | Avi.mar | N14, N16 | P | T | Ph | C_3_ | Eu | Salt-rec |
| 28 | *Beta vulgaris* L. | Bet.vul | N21 | A/BA | H | Th | C_3_ | Fac | Semi-suc |
| 29 | *Bienertia cycloptera* Bunge ex Boiss. | Bie.cyc | M2 | A | H | Th | C_4_ | Eu | Leaf-suc |
| 30 | *Bienertia sinuspersici* Akhani | Bie.sin | K, N15 | A | H | Th | C_4_ | Eu | Leaf-suc |
| 31 | *Bolboschoenus affinis* (Roth) Drobow | Bol.aff | U41, U82 | P | H | H | C_3_ | Eur | Non-suc |
| 32 | *Bolboschoenus glaucus* (Lam.) S. G. Sm. | Bol.gla | N13 | P | H | G | C_3_ | Eur | Non-suc |
| 33 | *Brassica tournefortii* Gouan | Bra.tou | N13 | A | H | Th | C_3_ | Eu | Non-suc |
| 34 | *Bromus tectorum* L. | Bro.tec | U110, U16, U52, U56, U6 | A | H | Th | C_3_ | Pes | Non-suc |
| 35 | *Calendula sancta* L. | Cal.san | N21 | A | H | Th | C_3_ | Pes | Non-suc |
| 36 | *Camphorosma monspeliaca* L. | Cam.mon | U18 | P | Sub-Sh | Ch | C_4_ | Fac | Semi-suc |
| 37 | *Capparis spinose* L. | Cap.spi | N3 | P | Sh | Ch | C_3_ | Fac | Semi-suc |
| 38 | *Carduus arabicus* Murray | Car.ara | U110, U19 | A | H | Th | C_3_ | Pes | Non-suc |
| 39 | *Caroxylon dendroides* (Pall.) Tzvel. (=*Salsola dendroides* Pall.)* | Car.den | U110, U111, U17, U19 | P | Sub-Sh | Ch | C_4_ | Eu | Leaf-suc |
| 40 | *Caroxylon imbricatum* (Forssk.) Moq. (*imbricata* Forssk.)* | Car.imb | N11, N15 | P | Sh | Ph | C_4_ | Eu | Leaf-suc |
| 41 | *Caroxylon nitrarium* (Pall.) Akhani & Roalson (=*Salsola nitraria* Pall.)* | Car.nit | U33, U35, U6 | A | H | Th | C_4_ | Eu | Leaf-suc |
| 42 | *Ceratocephalus falcatus* (L.) Pers. | Cer.fal | U16, U51, U52, U55 | A | H | Th | C_3_ | Pes | Non-suc |
| 43 | *Chenopodiastrum murale* (L.) S.Fuentes, Uotila & Borsch (=*Chenopodium murale* L.)* | Che.mur | N3 | A | H | Th | C_3_ | Pes | Non-suc |
| 44 | *Cirsium alatum* (S. G. Gmel) Bobrov | Cir.ala | U16 | P | H | H | C_3_ | Eu | Semi-suc |
| 45 | *Climacoptera crassa* (M. Bieb.) Botsch. (=*Salsola crassa* M. Bieb.) * | Cli.cra | U110, U21, U22, U31, U32, U33, U34, U35, U51, U6 | A | H | Th | C_4_ | Eu | Leaf-suc |
| 46 | *Climacoptera lanata* (Pall.) Botsch. (=*Salsola lanata* Pall.)* | Cli.Lan | M2 | A | H | Th | C_4_ | Eu | Leaf-suc |
| 47 | *Cocculus pendulus* (J. R. Forst. & G. Forst.) Diels | Coc.pen | N3 | P | WC | Ph | C_3_ | Pes | Non-suc |
| 48 | *Convolvulus lineatus* L. | Con.lin | U18 | P | H | H | C_3_ | Eu | Non-suc |
| 49 | *Crepis sancta* (L.) Babc. | Cre.san | U55, U6 | A | H | Th | C_3_ | Pes | Non-suc |
| 50 | *Cressa cretica* L. | Cre.cre | N21 | P | H | H | C_3_ | Eu | Salt-rec |
| 51 | *Cynanchum acutum* L. | Cyn.acu | U43 | P | H | H | C_3_ | Fac | Non-suc |
| 52 | *Descurainia sophia* (L.) Webb ex Prantl | Des.sop | U110, U16, U19, U51, U52, U54 | A | H | Th | C_3_ | Pes | Non-suc |
| 53 | *Emex spinose* (L.) Campd. | Eme.spi | N5 | A | H | Th | C_3_ | Fac | Semi-suc |
| 54 | *Eremopogon foveolatus* Stapf | Ere.fov | N15 | P | H | H | C_3_ | Fac | Non-suc |
| 55 | *Eremopyrum distans* (K.Koch) Nevski | Ere.dis | U11 | A | H | Th | C_3_ | Fac | Non-suc |
| 56 | *Eremopyrum triticeum* (Gaertn.) Nevski | Ere.tri | U18, U51, U53, U54 | A | H | Th | C_3_ | Fac | Non-suc |
| 57 | *Erodium cicutarium* (L.) L'Hér. | Ero.cic | U110, U111, U16, U17, U19, U6 | A | H | Th | C_3_ | Pes | Non-suc |
| 58 | *Erodium neuradifolium* Delile ex Godr. | Ero.neu | N13 | A | H | Th | C_3_ | Fac | Non-suc |
| 59 | *Erucaria hispanica* (L.) Druce | Eru.his | N5 | A | H | Th | C_3_ | Fac | Semi-suc |
| 60 | *Erysimum repandum* L. | Ery.rep | M2 | A | H | Th | C_3_ | Fac | Non-suc |
| 61 | *Erysimum sisymbrioides* C.A.Mey. | Ery.sis | U16 | A | H | Th | C_3_ | Fac | Non-suc |
| 62 | *Euclidium syriacum* (L.) W. T. Aiton | Euc.syr | U110, U19, U52 | A | H | Th | C_3_ | Fac | Non-suc |
| 63 | *Euphorbia heteradena* Jaub. & Spach | Eup.het | U24 | P | H | H | C_3_ | Pes | Non-suc |
| 64 | *Fagonia bruguieri* DC. | Fag.bru | N5 | P | H | Ch | C_3_ | Fac | Semi-suc |
| 65 | *Frankenia hirsuta* L. | Fra.hir | U11, U12 | P | Sub-Sh | Ch/H | C_3_ | Eu | Salt-rec |
| 66 | *Frankenia pulverulenta* L. | Fra.pul | N4 | A | H | Th | C_3_ | Eu | Salt-rec |
| 67 | *Fumaria asepala* Boiss. | Fum.ase | U55 | A | H | Th | C_3_ | Pes | Non-suc |
| 68 | *Galium spurium* L. | Gal.spu | U56 | A | H | Th | C_3_ | Pes | Non-suc |
| 69 | *Galium tricornutum* Dandy | Gal.tri | U16 | A | H | Th | C_3_ | Pes | Non-suc |
| 70 | *Galium verticillatum* Danthoine ex Lam. | Gal.ver | U110, U18 | A | H | Th | C_3_ | Pes | Non-suc |
| 71 | *Geranium lucidum* L. | Ger.luc | N3 | A | H | Th | C_3_ | Pes | Non-suc |
| 72 | *Grantia aucheri* Boiss. | Gra.auc | N5 | P | H | H | C_3_ | Pes | Semi-suc |
| 73 | *Gypsophila perfoliata* L. | Gyp.per | U16, U19 | P | H | H | C_3_ | Eu | Semi-suc |
| 74 | *Halimioine verrucifera* (M.Bieb.) Aellen (=*Atriplex verrucifera* M.Bieb.) * | Hal.ver | N21, U110, U12, U16, U18, U19, U34, U35 | P | Sub-Sh | Ch | C_3_ | Eu | Salt-rec |
| 75 | *Halimocnemis rarifolia* (K. Koch) Akhani (=*Halanthium rarifolium* C. Koch)* | Hal.rar | U11, U35, U6 | A | H | Th | C_4_ | Eu | Leaf-suc |
| 76 | *Halocharis sulphurea* (Moq.) Moq. | Hal.sul | N21 | A | H | Th | C_4_ | Eu | Leaf-suc |
| 77 | *Halocnemum strobilaceum* (Pall.) M. Bieb. | Hal.str | K, N4, U11, U12, U32, U35, U85 | P | Sh | Ch | C_3_ | Eu | Stem-suc |
| 78 | *Halopyrum mucronatum* Stapf | Hal.muc | N13, N11, N15 | P | H | H | C_4_ | Eu | Non-suc |
| 79 | *Helianthemum salicifolium* (L.) Mill. | Hel.sal | U111, U16, U17, U24 | A | H | Th | C_3_ | Pes | Non-suc |
| 80 | *Heliotropium bacciferum* Forssk. | Hel.bac | N12, N3 | P | Sub-Sh | Ch | C_3_ | Fac | Semi-suc |
| 81 | *Hippocrepis bisiliqua* (L.) Lassen | Hip.bis | N13 | A | H | Th | C_3_ | Fac | Non-suc |
| 82 | *Holosteum glutinosum* (M. Bieb.) Fisch. & C. A. Mey. | Hol.glu | U16, U55 | A | H | Th | C_3_ | Pes | Semi-suc |
| 83 | *Holosteum umbellatum* L. | Hol.umb | U16, U24, U52, U53, U6 | A | H | Th | C_3_ | Pes | Semi-suc |
| 84 | *Hordeum murinum subsp. Glaucum* (Steud.) Tzvelev | Hor.mur | U6, U110, U17, U18, U19, U24, U51 | A | H | Th | C_3_ | Fac | Non-suc |
| 85 | *Hornungia procumbens* Hayek. (=*Hymenolobus procumbens* (L.) Hedge & Lamond)* | Hor.pro | U17, U6 | A | H | Th | C_3_ | Eu | Non-suc |
| 86 | *Hypecoum pendulum* L. | Hyp.pen | U110, U52 | A | H | Th | C_3_ | Pes | Non-suc |
| 87 | *Inula aucheriana* DC. | Inu.auc | U14 | P | H | H | C_3_ | Eu | Non-suc |
| 88 | *Iris pseudocaucasica* Grossh. | Iri.pse | U16 | P | H | H | C_3_ | Pes | Non-suc |
| 89 | *Iris spuria* L. | Iri.spu | U16 | P | H | H | C_3_ | Eur | Non-suc |
| 90 | *Juncus heldreichianus* T. Marsson ex Parl. | Jun.hel | U12, U13, U14, U15, U16, U42 | P | H | H | C_3_ | Eur | Non-suc |
| 91 | *Koelpinia linearis* Pall. | Koe.lin | U17 | A | H | Th | C_3_ | Fac | Non-suc |
| 92 | *Lamium amplexicaule* L. | Lam.amp | U16, U52 | A | H | Th | C_3_ | Pes | Non-suc |
| 93 | *Lappula sp.* | Lap.sp. | U52 | A | H | Th | C_3_ | Pes | Non-suc |
| 94 | *Lappula spinocarpos* (Forssk.) Asch. ex Kuntze | Lap.spi | U24 | A | H | Th | C_3_ | Pes | Non-suc |
| 95 | *Lasiopogon muscoides* DC. | Las.mus | N5 | A | H | Th | C_3_ | Pes | Non-suc |
| 96 | *Launaea mucronata* (Forssk.) Muschl. | Lau.muc | N12 | A | H | Th | C_3_ | Fac | Non-suc |
| 97 | *Launaea procumbens* (Roxb.) Amin | Lau.pro | N11 | P | H | Ch | C_3_ | Fac | Non-suc |
| 98 | *Lepidium cartilagineum* (J. C. Mayer) Thell. | Lep.car | U12, U16 | P | H | H | C_3_ | Eu | Semi-suc |
| 99 | *Lepidium draba* L. | Lep.dra | U110, U14, U16, U19, U53 | P | H | H | C_3_ | Fac | Non-suc |
| 100 | *Lepidium perfoliatum* L. | Lep.per | M1, U11, U54 | A | H | Th | C_3_ | Fac | Non-suc |
| 101 | *Lepidium vesicarium* L. | Lep.ves | U52 | A | H | Th | C_3_ | Fac | Non-suc |
| 102 | *Leptaleum filifolium* DC. | Lep.fil | U110 | A | H | Th | C_3_ | Fac | Semi-suc |
| 103 | *Limonium axillare* (Forssk.) Kuntze | Lim.axi | N15 | P | Sub-Sh | H | C_3_ | Eu | Salt-rec |
| 104 | *Limonium failachicum* Erben & Mucina | Lim.fai | K, N15 | P | Sub-Sh | H | C_3_ | Eu | Salt-rec |
| 105 | *Limonium meyeri* Kuntze | Lim.mey | U12, U16 | P | H | H | C_3_ | Eu | Salt-rec |
| 106 | *Linaria simplex* DC. | Lin.sim | U6 | A | H | Th | C_3_ | Pes | Non-suc |
| 107 | *Lithospermum arvense* L. | Lit.arv | U24 | A | H | Th | C_3_ | Pes | Non-suc |
| 108 | *Lotus halophilus* Boiss. & Spruner | Lot.hal | N11 | A | H | Th | C_3_ | Eu | Non-suc |
| 109 | *Lycium ruthenicum* Murray | Lyc.rut | U17 | P | Sh | Nano-Ph | C_3_ | Fac | Semi-suc |
| 110 | *Lycium shawii* Roem. & Schult. | Lyc.sha | N22 | P | Sh | Nano-Ph | C_3_ | Fac | Semi-suc |
| 111 | *Malcolmia africana* (L.) W. T. Aiton | Mal.afr | U19, U6 | A | H | Th | C_3_ | Fac | Non-suc |
| 112 | *Malcolmia strigosa* Boiss. | Mal.str | U110 | A | H | Th | C_3_ | Pes | Non-suc |
| 113 | *Malva parviflora* L. | Mal.par | N3 | A | H | Th | C_3_ | Fac | Non-suc |
| 114 | *Medicago polymorpha* L. | Med.pol | N21 | A | H | Th | C_3_ | Fac | Non-suc |
| 115 | *Mesembryanthemum nodiflorum* L. | Mes.nod | N3, N4 | A | H | Th | C_3_/CAM | Eu | Stem-Leaf-suc |
| 116 | *Neotorularia torulosa* (Desf.) Hedge & J.Léonard | Neo.tor | U111, U18 | A | H | Th | C_3_ | Fac | Non-suc |
| 117 | *Nitraria schoberi* L. | Nit.sch | M1, U12 | P | Sh | Ch | C_3_ | Eu | Semi-suc |
| 118 | *Nonnea caspica* (Willd.) G. Don | Non.cas | U52 | A | H | Th | C_3_ | Pes | Non-suc |
| 119 | *Ochradenus baccatus* Delile | Och.bac | N3 | P | Sh | Ch | C_3_ | Fac | Semi-suc |
| 120 | *Olimarabidopsis pumila* (Stephan) Al-Shehbaz & al. | Oli.pum | U16, U19, U51, U54, U6 | A | H | Th | C_3_ | Fac | Non-suc |
| 121 | *Papaver argemone* L. | Pap.arg | U24 | A | H | Th | C_3_ | Pes | Non-suc |
| 122 | *Parietaria alsinifolia* Delile | Par.als | N3 | A | H | Th | C_3_ | Pes | Non-suc |
| 123 | *Peganum harmala* L. | Peg.har | U110, U17, U24 | P | H | H | C_3_ | Fac | Non-suc |
| 124 | *Petrosimonia brachiata* (Pall.) Bunge | Pet.bra | U11 | A | H | Th | C_4_ | Eu | Leaf-suc |
| 125 | *Petrosimonia glauca* (Pall.) Bunge | Pet.gla | M2 | A | H | Th | C_4_ | Eu | Leaf-suc |
| 126 | *Phragmites australis* (Cav.) Steud. | Phr.aus | U12, U14, U16, U83 | P | H | H | C_3_ | Eur | Non-suc |
| 127 | *Plantago amplexicaulis* Cav. | Pla.amp | N21 | A | H | Th | C_3_ | Pes | Non-suc |
| 128 | *Plantago boissieri* Hausskn. & Bornm. ex Bornm. | Pla.boi | N12 | A | H | Th | C_3_ | Eu | Non-suc |
| 129 | *Plantago coronopus* L. | Pla.cor | N4 | A | H | Th | C_3_ | Fac | Non-suc |
| 130 | *Plantago maritima* L. | Pla.mar | U18 | P | H | H | C_3_ | Eu | Leaf-suc |
| 131 | *Poa bulbosa* L. | Poa.bul | U110, U52 | P | H | H | C_3_ | Pes | Non-suc |
| 132 | *Polygonum patulum* M. Bieb. | Pol.pat | U55, U6 | A | H | Th | C_3_ | Fac | Non-suc |
| 133 | *Psylliostachys spicata* (Willd.) Nevski | Psy.spi | N4 | A | H | Th | C_3_ | Eu | Non-suc |
| 134 | *Puccinellia bulbosa* (Grossh.) Grossh. | Puc.bul | U16 | P | H | H | C_3_ | Eu | Non-suc |
| 135 | *Puccinellia distans* Parl. | Puc.dis | M1 | P | H | H | C_3_ | Eu | Non-suc |
| 136 | *Pulicaria arabica* (L.) Cass. | Pul.ara | N3 | P | Sub-Sh | Ch | C_3_ | Pes | Non-suc |
| 137 | *Reaumuria alternifolia* (Labill.) Britten | Rea.alt | M1 | P | Su | Ch | C_3_ | Eu | Salt-rec |
| 138 | *Reichardia orientalis* Hochr. | Rei.ori | N5 | A | H | Th | C_3_ | Pes | Non-suc |
| 139 | *Rochelia disperma* Hochr. | Roc.dis | U24 | A | H | Th | C_3_ | Pes | Non-suc |
| 140 | *Roemeria hybrid* DC. | Roe.hyb | U110, U24, U52, U6 | A | H | Th | C_3_ | Pes | Non-suc |
| 141 | *Rumex vesicarius* L. | Rum.ves | N5 | A | H | Th | C_3_ | Pes | Non-suc |
| 142 | *Salicornia iranica* Akhani* | Sal.ira | U32, U41, U81, U6 | A | H | Th | C_3_ | Eu | Stem-suc |
| 143 | *Salicornia iranica* subsp. *sinus-persica* (Akhani) Chatrenoor & Akhani* | Sal.sin | K, N14 | A | H | Th | C_3_ | Eu | Stem-suc |
| 144 | *Soda drummondii* (Ulbr.) Akhani (= *Salsola drummondii* Ulbr.)* | Sod.dru | N5 | P | Sh | Ch | C_4_ | Eu | Leaf-suc |
| 145 | *Soda inermis* Fourr. (= *Salsola soda* L.)* | Sod.ine | U7 | A | H | Th | C_4_ | Eu | Leaf-suc |
| 146 | *Saussurea salsa* (Pall. ex Pall.) Spreng. | Sau.sal | U14, U15 | P | H | H | C_3_ | Eu | Semi-suc |
| 147 | *Sclerochloa dura* (L.) P. Beauv. | Scl.dur | U110, U11, U16, U54 | A | H | Th | C_3_ | Fac | Non-suc |
| 148 | *Scorpiurus muricatus* L. | Sco.mur | N21 | A | H | Th | C_3_ | Fac | Non-suc |
| 149 | *Scorzonera laciniata* Jacq. | Sco.lac | U16, U18, U19 | BA | H | H | C_3_ | Fac | Non-suc |
| 150 | *Scorzonera parviflora* Jacq. | Sco.par | U16 | P | H | H | C_3_ | Pes | Non-suc |
| 151 | *Senecio glaucus* L. | Sen.gla | N12, U110, U111, U16, U17, U19, U24, U6 | A | H | Th | C_3_ | Fac | Semi-suc |
| 152 | *Senecio vernalis* Franch. | Sen.ver | U51, U54 | A | H | Th | C_3_ | Fac | Semi-suc |
| 153 | *Silene coniflora* Nees ex Otth | Sil.coi | U24 | A | H | Th | C_3_ | Pes | Non-suc |
| 154 | *Silene conoidea* L. | Sil.con | U16, U17, U18, U24 | A | H | Th | C_3_ | Pes | Non-suc |
| 155 | *Sisymbrium septulatum* DC. | Sis.sep | U110, U111, U17, U19, U51 | A | H | Th | C_3_ | Pes | Non-suc |
| 156 | *Solanum incanum* L. | Sol.inc | N3 | P | Sh | Ch | C_3_ | Pes | Non-suc |
| 157 | *Sonchus asper* (L.) Hill | Son.asp | N5 | BA | H | Th | C_3_ | Fac | Non-suc |
| 158 | *Spergularia diandra* (Guss.) Boiss. | Spe.dia | N4, U24 | A | H | Th | C_3_ | Eu | Leaf-suc |
| 159 | *Spergularia marina* (L.) Griseb. | Spe.mar | U85 | A | H | Th | C_3_ | Eu | Leaf-suc |
| 160 | *Spinacia tetrandra* M. Bieb. | Spi.tet | U110, U19 | A | H | Th | C_3_ | Fac | Non-suc |
| 161 | *Sporobolus arabicus* Boiss. | Spo.ara | N15 | P | H | H | C_4_ | Eu | Salt-rec |
| 162 | *Stipa capensis* Thunb. | Sti.cap | N3 | A | H | Th | C_3_ | Pes | Non-suc |
| 163 | *Stipa hohenackeriana* Trin. & Rupr. | Sti.hoh | M1, U111 | P | H | H | C_3_ | Pes | Non-suc |
| 164 | *Suaeda aegyptiaca* (Hasselq.) Zohary | Sua.aeg | N4 | A | H | Th | C_4_ | Eu | Leaf-suc |
| 165 | *Suaeda altissima* (L.) Pall. | Sua.alt | U11, U21, U22, U23, U24, U31, U53, U6, U55 | A | H | Th | C_4_ | Eu | Leaf-suc |
| 166 | *Suaeda fruticosa* Forssk. | Sua.fru | K, N15, N3 | P | Sh | Ch | C_4_ | Eu | Leaf-suc |
| 167 | *Suaeda gracilis* Moq.* | Sua.gra | U31, U32, U6 | A | H | Th | C_4_ | Eu | Leaf-suc |
| 168 | *Suaeda heterophylla* (Kar. & Kir.) Boiss. | Sua.het | U6 | A | H | Th | C_3_ | Eu | Leaf-suc |
| 169 | *Suaeda iranshahri* Akhani & Freitag* | Sua.ira | K | A/BA | H | Th | C_3_ | Eu | Leaf-suc |
| 170 | *Suaeda khalijefarsica* Akhani* | Sua.kha | K | A | H | Th | C_4_ | Eu | Leaf-suc |
| 171 | *Tamarix androssowii* Litv. | Tam.and | U6 | P | Sh | Ph | C_3_ | Eu | Salt-rec |
| 172 | *Tamarix octandra* Bunge | Tam.oct | U56 | P | Sh | Ph | C_3_ | Eu | Salt-rec |
| 173 | *Tamarix pycnocarpa* DC. | Tam.pyc | N4 | P | Sh | Ph | C_3_ | Eu | Salt-rec |
| 174 | *Taraxacum sp.* | Tar.sp. | U14, U16 | P | H | H | C_3_ | Fac | Non-suc |
| 175 | *Tetradiclis tenella* Litv. | Tet.ten | U110 | A | H | Th | C_3_ | Eu | Leaf-suc |
| 176 | *Thesium compressum* Boiss. & Heldr | The.com | U16 | BA | H | Th | C_3_ | Eu | Leaf-suc |
| 177 | *Tragopogon graminifolius* DC. | Tra.gra | U16 | P | H | H | C_3_ | Pes | Non-suc |
| 178 | *Trigonella arcuate* Boiss. & C.I.Blanche | Tri.arc | U110, U17, U19 | A | H | Th | C_3_ | Pes | Non-suc |
| 179 | *Trigonella coerulescens* Boiss. & C.I.Blanche | Tri.coe | U24 | A | H | Th | C_3_ | Pes | Non-suc |
| 180 | *Tripleurospermum parviflorum* (Willd.) Pobed. | Tri.par | U110, U16, U17, U18, U19, U24,U54 | A | H | Th | C_3_ | Pes | Non-suc |
| 181 | *Typha grossheimii* Pobed. | Typ.gro | U42 | P | H | He | C_3_ | Eur | Non-suc |
| 182 | *Valerianella plagiostephana* Fisch. & C.A.Mey. | Val.pla | U52 | A | H | Th | C_3_ | Pes | Non-suc |
| 183 | *Veronica campylopoda* Boiss. | Ver.cam | U110, U16, U52 | A | H | Th | C_3_ | Pes | Non-suc |
| 184 | *Veronica persica* Poir. | Ver.per | U56 | A | H | Th | C_3_ | Pes | Non-suc |
| 185 | *Viola occulta* Hort.Argent. ex Otto | Vio.occ | U56 | A | H | Th | C_3_ | Pes | Non-suc |
| 186 | *Vulpia persica* (Boiss. & Buhse) V.I.Krecz. & Bobr. | Vul.per | U19 | A | H | Th | C_3_ | Pes | Non-suc |
| 187 | *Ziziphus nummularia* (Burm. f.) Wight & Arn. | Ziz.num | N3 | P | T | Ph | C_3_ | Fac | Non-suc |
| 188 | *Zygophyllum fabago* L. | Zyg.fab | U16, U53 | P | H | H | C_3_ | Fac | Semi-suc |

Supporting Information to the paper Matinzadeh, Z. Functional structure of plant communities along salinity gradients in Iranian salt marshes. *Plant-Environment Interactions*.

Appendix S3. Plant species in 48 studied plots with their cover percentage (%).

| **Species/Plot** | **U11** | **U12** | **U13** | **U14** | **U15** | **U16** | **U17** | **U18** | **U19** | **U110** | **U111** | **U21** | **U22** | **U23** | **U24** | **U31** | **U32** | **U33** | **U34** | **U35** | **U41** | **U42** | **U43** | **U51** | **U52** | **U53** | **U54** | **U55** | **U56** | **U6** | **U7** | **U81** | **U82** | **U83** | **N11** | **N12** | **N13** | **N14** | **N15** | **N16** | **N21** | **N22** | **N3** | **N4** | **N5** | **K** | **M1** | **M2** |
| --- | --- | --- | --- | --- | --- | --- | --- | --- | --- | --- | --- | --- | --- | --- | --- | --- | --- | --- | --- | --- | --- | --- | --- | --- | --- | --- | --- | --- | --- | --- | --- | --- | --- | --- | --- | --- | --- | --- | --- | --- | --- | --- | --- | --- | --- | --- | --- | --- |
| Ach.ten | . | . | . | . | . | 4.12 | . | 10.7 | 4.5 | 2 | . | . | . | . | . | . | . | . | . | . | . | . | . | . | . | . | . | . | . | . | . | . | . | . | . | . | . | . | . | . | . | . | . | . | . | . | . | . |
| Aci.gra | . | . | . | . | . | . | . | . | . | . | . | . | . | . | . | . | . | . | . | . | . | . | . | . | 0.5 | . | . | . | . | . | . | . | . | . | . | . | . | . | . | . | . | . | . | . | . | . | . | . |
| Ado.ann | . | . | . | . | . | 0.1 | . | . | 0.23 | 0.36 | . | . | . | . | . | . | . | . | . | . | . | . | . | . | . | . | . | . | 0.5 | . | . | . | . | . | . | . | . | . | . | . | . | . | . | . | . | . | . | . |
| Ael.lag | . | . | . | . | . | . | . | . | . | . | . | . | . | . | . | . | . | . | . | . | . | . | . | . | . | . | . | . | . | . | . | . | . | . | . | . | . | . | . | . | . | 6.8 | . | . | . | . | . | . |
| Ael.lit | . | 15.3 | . | . | 3.6 | 3.2 | . | 1.7 | 1.1 | 1.2 | . | . | . | . | . | . | . | . | . | . | . | . | . | . | . | . | . | . | . | 71 | . | . | . | . | . | . | . | . | . | . | . | . | . | . | . | . | . | . |
| Alh.mau | . | . | . | . | . | 6 | 3 | . | 21.8 | . | . | . | . | . | 72 | . | . | . | 27.5 | . | . | . | 22.5 | . | . | . | . | . | . | . | . | . | . | . | . | . | . | . | . | . | . | . | . | . | . | . | . | . |
| Alo.myo | . | . | . | . | . | . | . | . | . | . | . | . | . | . | . | . | . | . | . | . | . | . | . | . | . | 5.3 | . | . | . | . | . | . | . | . | . | . | . | . | . | . | . | . | . | . | . | . | . | . |
| Aly.das | . | . | . | . | . | . | . | . | . | . | . | . | . | . | . | . | . | . | . | . | . | . | . | . | 0.5 | . | . | . | . | . | . | . | . | . | . | . | . | . | . | . | . | . | . | . | . | . | . | . |
| Aly.des | . | . | . | . | . | . | . | 2 | . | . | . | . | . | . | . | . | . | . | . | . | . | . | . | . | 0.5 | . | . | . | . | . | . | . | . | . | . | . | . | . | . | . | . | . | . | . | . | . | . | . |
| Aly.lin | . | . | . | . | . | 1 | . | . | . | . | . | . | . | . | . | . | . | . | . | . | . | . | . | 0.42 | . | . | . | . | . | . | . | . | . | . | . | . | . | . | . | . | . | . | . | . | . | . | 0.5 | . |
| And.max | . | . | . | . | . | 0.53 | . | . | . | . | . | . | . | . | . | . | . | . | . | . | . | . | . | . | . | 0.5 | . | . | . | . | . | . | . | . | . | . | . | . | . | . | . | . | . | . | . | . | . | . |
| Ant.aus | . | . | . | . | . | . | . | . | . | . | . | . | . | . | . | . | . | . | . | . | . | . | . | . | 0.5 | . | . | . | . | . | . | . | . | . | . | . | . | . | . | . | . | . | . | . | . | . | . | . |
| Are.lep | . | . | . | . | . | . | . | . | . | . | . | . | . | . | . | . | . | . | . | . | . | . | . | . | . | . | . | . | . | 0.6 | . | . | . | . | . | . | . | . | . | . | . | . | . | . | . | . | . | . |
| Arn.dec | . | . | . | . | . | . | . | . | . | . | . | . | . | . | . | . | . | . | . | . | . | . | . | . | . | . | . | . | . | . | . | . | . | . | . | . | . | . | . | . | . | . | . | . | . | . | 1.1 | . |
| Art.mac | . | . | . | . | . | . | . | . | . | . | . | . | . | . | . | . | . | . | . | . | . | . | . | . | . | . | . | . | . | . | . | . | . | . | . | . | . | . | . | 38 | . | . | . | . | . | . | . | . |
| Art.sp.1 | . | . | . | . | . | . | . | . | . | . | . | . | . | . | . | . | . | . | . | . | . | . | . | . | . | . | . | . | . | . | . | . | . | . | . | . | . | . | . | . | . | . | . | . | . | . | 29 | . |
| Art.sp.2 | . | . | . | . | . | . | . | . | . | . | . | . | . | . | . | . | . | . | . | . | . | . | . | . | 0.5 | . | . | . | . | . | . | . | . | . | . | . | . | . | . | . | . | . | . | . | . | . | . | . |
| Art.spi | . | . | . | . | . | . | . | . | . | 5.25 | 17 | . | . | . | . | . | . | . | . | . | . | . | . | . | . | . | . | . | . | . | . | . | . | . | . | . | . | . | . | . | . | . | . | . | . | . | . | . |
| Asp.pro | . | . | . | . | . | 0.75 | . | . | . | . | . | . | . | . | . | . | . | . | . | . | . | . | . | . | 0.5 | . | . | . | . | 0.7 | . | . | . | . | . | . | . | . | . | . | . | . | . | . | . | . | . | . |
| Asp.ver | . | . | . | . | . | 0.3 | . | . | . | . | . | . | . | . | . | . | . | . | . | . | . | . | . | . | . | . | . | . | . | . | . | . | . | . | . | . | . | . | . | . | . | . | . | . | . | . | . | . |
| Ast.cre | . | . | . | . | . | 0.5 | . | . | 0.5 | 0.75 | . | . | . | . | . | . | . | . | . | . | . | . | . | . | . | . | . | . | . | . | . | . | . | . | . | . | . | . | . | . | . | . | . | . | 4.4 | . | . | . |
| Atr.can | . | . | . | . | . | . | . | . | . | . | . | . | . | . | . | . | . | . | . | . | . | . | . | . | . | . | . | . | . | . | . | . | . | . | . | . | . | . | . | . | . | . | . | . | . | . | 12 | . |
| Atr.leu | . | . | . | . | . | 15 | 0.5 | . | 2.25 | 3.6 | . | . | . | . | . | . | . | . | . | . | . | . | . | . | . | . | . | . | . | . | . | . | . | . | . | . | . | . | . | . | . | . | . | . | . | . | 3.6 | . |
| Atr.mic | . | . | . | . | . | . | . | . | . | . | . | . | . | . | . | . | . | . | . | . | . | . | . | . | . | . | . | 1 | . | . | . | . | . | . | . | . | . | . | . | . | . | . | . | . | . | . | . | . |
| Atr.tat | . | . | . | . | . | . | . | . | . | . | . | . | . | . | . | 4 | 15 | 3 | . | . | . | . | . | . | . | . | . | . | . | . | . | . | . | . | . | . | . | . | . | . | . | . | . | . | . | . | . | . |
| Ave.lud | . | . | . | . | . | . | . | . | . | . | . | . | . | . | . | . | . | . | . | . | . | . | . | . | . | . | . | . | . | . | . | . | . | . | . | . | . | . | . | . | . | . | . | . | 0.7 | . | . | . |
| Avi.mar | . | . | . | . | . | . | . | . | . | . | . | . | . | . | . | . | . | . | . | . | . | . | . | . | . | . | . | . | . | . | . | . | . | . | . | . | . | . | 100 | . | . | . | . | . | . | . | . | . |
| Bet.vul | . | . | . | . | . | . | . | . | . | . | . | . | . | . | . | . | . | . | . | . | . | . | . | . | . | . | . | . | . | . | . | . | . | . | . | . | . | . | . | . | 22 | . | . | . | . | . | . | . |
| Bie.cyc | . | . | . | . | . | . | . | . | . | . | . | . | . | . | . | . | . | . | . | . | . | . | . | . | . | . | . | . | . | . | . | . | . | . | . | . | . | . | . | . | . | . | . | . | . | . | . | 7 |
| Bie.sin | . | . | . | . | . | . | . | . | . | . | . | . | . | . | . | . | . | . | . | . | . | . | . | . | . | . | . | . | . | . | . | . | . | . | . | . | . | 2.6 | . | . | . | . | . | . | . | 3.2 | . | . |
| Bol.aff | . | . | . | . | . | . | . | . | . | . | . | . | . | . | . | . | . | . | . | . | 30 | . | . | . | . | . | . | . | . | . | . | . | 86 | . | . | . | . | . | . | . | . | . | . | . | . | . | . | . |
| Bol.gla | . | . | . | . | . | . | . | . | . | . | . | . | . | . | . | . | . | . | . | . | . | . | . | . | . | . | . | . | . | . | . | . | . | . | . | . | 63 | . | . | . | . | . | . | . | . | . | . | . |
| Bra.tou | . | . | . | . | . | . | . | . | . | . | . | . | . | . | . | . | . | . | . | . | . | . | . | . | . | . | . | . | . | . | . | . | . | . | . | . | 0.6 | . | . | . | . | . | . | . | . | . | . | . |
| Bro.tec | . | . | . | . | . | 5 | . | . | . | 7 | . | . | . | . | . | . | . | . | . | . | . | . | . | . | 15 | . | . | . | 62.5 | 4 | . | . | . | . | . | . | . | . | . | . | . | . | . | . | . | . | . | . |
| Cal.san | . | . | . | . | . | . | . | . | . | . | . | . | . | . | . | . | . | . | . | . | . | . | . | . | . | . | . | . | . | . | . | . | . | . | . | . | . | . | . | . | 0.9 | . | . | . | . | . | . | . |
| Cam.mon | . | . | . | . | . | . | . | 12 | . | . | . | . | . | . | . | . | . | . | . | . | . | . | . | . | . | . | . | . | . | . | . | . | . | . | . | . | . | . | . | . | . | . | . | . | . | . | . | . |
| Cap.spi | . | . | . | . | . | . | . | . | . | . | . | . | . | . | . | . | . | . | . | . | . | . | . | . | . | . | . | . | . | . | . | . | . | . | . | . | . | . | . | . | . | . | 2 | . | . | . | . | . |
| Car.ara | . | . | . | . | . | . | . | . | 0.5 | 1 | . | . | . | . | . | . | . | . | . | . | . | . | . | . | . | . | . | . | . | . | . | . | . | . | . | . | . | . | . | . | . | . | . | . | . | . | . | . |
| Car.den | . | . | . | . | . | . | 4.2 | . | 0.5 | 25 | 0.75 | . | . | . | . | . | . | . | . | . | . | . | . | . | . | . | . | . | . | . | . | . | . | . | . | . | . | . | . | . | . | . | . | . | . | . | . | . |
| Car.imb | . | . | . | . | . | . | . | . | . | . | . | . | . | . | . | . | . | . | . | . | . | . | . | . | . | . | . | . | . | . | . | . | . | . | 6.2 | . | . | 1.8 | . | . | . | . | . | . | . | . | . | . |
| Car.nit | . | . | . | . | . | . | . | . | . | . | . | . | . | . | . | . | . | 4 | . | 2.16 | . | . | . | . | . | . | . | . | . | 2 | . | . | . | . | . | . | . | . | . | . | . | . | . | . | . | . | . | . |
| Cer.fal | . | . | . | . | . | 0.1 | . | . | . | . | . | . | . | . | . | . | . | . | . | . | . | . | . | 0.1 | 18.4 | . | . | 11.25 | . | . | . | . | . | . | . | . | . | . | . | . | . | . | . | . | . | . | . | . |
| Che.mur | . | . | . | . | . | . | . | . | . | . | . | . | . | . | . | . | . | . | . | . | . | . | . | . | . | . | . | . | . | . | . | . | . | . | . | . | . | . | . | . | . | . | 0.8 | . | . | . | . | . |
| Cir.ala | . | . | . | . | . | 4 | . | . | . | . | . | . | . | . | . | . | . | . | . | . | . | . | . | . | . | . | . | . | . | . | . | . | . | . | . | . | . | . | . | . | . | . | . | . | . | . | . | . |
| Cli.cra | . | . | . | . | . | . | . | . | . | 2 | . | 89 | 13 | . | . | 28.75 | 17.5 | 37.5 | 9 | 3.6 | . | . | . | 30 | . | . | . | . | . | 5 | . | . | . | . | . | . | . | . | . | . | . | . | . | . | . | . | . | . |
| Cli.lan | . | . | . | . | . | . | . | . | . | . | . | . | . | . | . | . | . | . | . | . | . | . | . | . | . | . | . | . | . | . | . | . | . | . | . | . | . | . | . | . | . | . | . | . | . | . | . | 0.6 |
| Coc.pen | . | . | . | . | . | . | . | . | . | . | . | . | . | . | . | . | . | . | . | . | . | . | . | . | . | . | . | . | . | . | . | . | . | . | . | . | . | . | . | . | . | . | 2 | . | . | . | . | . |
| Con.lin | . | . | . | . | . | . | . | 34 | . | . | . | . | . | . | . | . | . | . | . | . | . | . | . | . | . | . | . | . | . | . | . | . | . | . | . | . | . | . | . | . | . | . | . | . | . | . | . | . |
| Cre.cre | . | . | . | . | . | . | . | . | . | . | . | . | . | . | . | . | . | . | . | . | . | . | . | . | . | . | . | . | . | . | . | . | . | . | . | . | . | . | . | . | 3.8 | . | . | . | . | . | . | . |
| Cre.san | . | . | . | . | . | . | . | . | . | . | . | . | . | . | . | . | . | . | . | . | . | . | . | . | . | . | . | 0.5 | . | 0.5 | . | . | . | . | . | . | . | . | . | . | . | . | . | . | . | . | . | . |
| Cyn.acu | . | . | . | . | . | . | . | . | . | . | . | . | . | . | . | . | . | . | . | . | . | . | 42.5 | . | . | . | . | . | . | . | . | . | . | . | . | . | . | . | . | . | . | . | . | . | . | . | . | . |
| Des.sop | . | . | . | . | . | 1.62 | . | . | 1.3 | 0.5 | . | . | . | . | . | . | . | . | . | . | . | . | . | 10.6 | 5 | . | 63 | . | . | . | . | . | . | . | . | . | . | . | . | . | . | . | . | . | . | . | . | . |
| Eme.spi | . | . | . | . | . | . | . | . | . | . | . | . | . | . | . | . | . | . | . | . | . | . | . | . | . | . | . | . | . | . | . | . | . | . | . | . | . | . | . | . | . | . | . | . | 1.12 | . | . | . |
| Ere.dis | 0.42 | . | . | . | . | . | . | . | . | . | . | . | . | . | . | . | . | . | . | . | . | . | . | . | . | . | . | . | . | . | . | . | . | . | . | . | . | . | . | . | . | . | . | . | . | . | . | . |
| Ere.fov | . | . | . | . | . | . | . | . | . | . | . | . | . | . | . | . | . | . | . | . | . | . | . | . | . | . | . | . | . | . | . | . | . | . | . | . | . | 20 | . | . | . | . | . | . | . | . | . | . |
| Ere.tri | . | . | . | . | . | . | . | 5 | . | . | . | . | . | . | . | . | . | . | . | . | . | . | . | 0.5 | . | 21.6 | 23.3 | . | . | . | . | . | . | . | . | . | . | . | . | . | . | . | . | . | . | . | . | . |
| Ero.cic | . | . | . | . | . | 0.55 | 1.15 | . | 0.5 | 2 | 10 | . | . | . | . | . | . | . | . | . | . | . | . | . | . | . | . | . | . | 2 | . | . | . | . | . | . | . | . | . | . | . | . | . | . | . | . | . | . |
| Ero.neu | . | . | . | . | . | . | . | . | . | . | . | . | . | . | . | . | . | . | . | . | . | . | . | . | . | . | . | . | . | . | . | . | . | . | . | . | 5.6 | . | . | . | . | . | . | . | . | . | . | . |
| Eru.hys | . | . | . | . | . | . | . | . | . | . | . | . | . | . | . | . | . | . | . | . | . | . | . | . | . | . | . | . | . | . | . | . | . | . | . | . | . | . | . | . | . | . | . | . | 0.5 | . | . | . |
| Ery.rep | . | . | . | . | . | . | . | . | . | . | . | . | . | . | . | . | . | . | . | . | . | . | . | . | . | . | . | . | . | . | . | . | . | . | . | . | . | . | . | . | . | . | . | . | . | . | . | 0.9 |
| Ery.sis | . | . | . | . | . | 1 | . | . | . | . | . | . | . | . | . | . | . | . | . | . | . | . | . | . | . | . | . | . | . | . | . | . | . | . | . | . | . | . | . | . | . | . | . | . | . | . | . | . |
| Euc.syr | . | . | . | . | . | . | . | . | 0.5 | 5.5 | . | . | . | . | . | . | . | . | . | . | . | . | . | . | 0.5 | . | . | . | . | . | . | . | . | . | . | . | . | . | . | . | . | . | . | . | . | . | . | . |
| Eup.het | . | . | . | . | . | . | . | . | . | . | . | . | . | . | 0.5 | . | . | . | . | . | . | . | . | . | . | . | . | . | . | . | . | . | . | . | . | . | . | . | . | . | . | . | . | . | . | . | . | . |
| Fag.bru | . | . | . | . | . | . | . | . | . | . | . | . | . | . | . | . | . | . | . | . | . | . | . | . | . | . | . | . | . | . | . | . | . | . | . | . | . | . | . | . | . | . | . | . | 3 | . | . | . |
| Fra.hir | 2 | 1 | . | . | . | . | . | . | . | . | . | . | . | . | . | . | . | . | . | . | . | . | . | . | . | . | . | . | . | . | . | . | . | . | . | . | . | . | . | . | . | . | . | . | . | . | . | . |
| Fra.pul | . | . | . | . | . | . | . | . | . | . | . | . | . | . | . | . | . | . | . | . | . | . | . | . | . | . | . | . | . | . | . | . | . | . | . | . | . | . | . | . | . | . | . | 0.5 | . | . | . | . |
| Fum.ase | . | . | . | . | . | . | . | . | . | . | . | . | . | . | . | . | . | . | . | . | . | . | . | . | . | . | . | 1 | . | . | . | . | . | . | . | . | . | . | . | . | . | . | . | . | . | . | . | . |
| Gal.spu | . | . | . | . | . | . | . | . | . | . | . | . | . | . | . | . | . | . | . | . | . | . | . | . | . | . | . | . | 14.4 | . | . | . | . | . | . | . | . | . | . | . | . | . | . | . | . | . | . | . |
| Gal.tri | . | . | . | . | . | 1.83 | . | . | . | . | . | . | . | . | . | . | . | . | . | . | . | . | . | . | . | . | . | . | . | . | . | . | . | . | . | . | . | . | . | . | . | . | . | . | . | . | . | . |
| Gal.ver | . | . | . | . | . | . | . | 16.5 | . | 1 | . | . | . | . | . | . | . | . | . | . | . | . | . | . | . | . | . | . | . | . | . | . | . | . | . | . | . | . | . | . | . | . | . | . | . | . | . | . |
| Ger.luc | . | . | . | . | . | . | . | . | . | . | . | . | . | . | . | . | . | . | . | . | . | . | . | . | . | . | . | . | . | . | . | . | . | . | . | . | . | . | . | . | . | . | 1.1 | . | . | . | . | . |
| Gra.auc | . | . | . | . | . | . | . | . | . | . | . | . | . | . | . | . | . | . | . | . | . | . | . | . | . | . | . | . | . | . | . | . | . | . | . | . | . | . | . | . | . | . | . | . | 7.8 | . | . | . |
| Gyp.per | . | . | . | . | . | 0.5 | . | . | 0.75 | . | . | . | . | . | . | . | . | . | . | . | . | . | . | . | . | . | . | . | . | . | . | . | . | . | . | . | . | . | . | . | . | . | . | . | . | . | . | . |
| Hal.muc | . | . | . | . | . | . | . | . | . | . | . | . | . | . | . | . | . | . | . | . | . | . | . | . | . | . | . | . | . | . | . | . | . | . | 18.6 | . | 4 | . | . | . | . | . | . | . | . | . | . | . |
| Hal.rar | 5.8 | . | . | . | . | . | . | . | . | . | . | . | . | . | . | . | . | . | . | 13.6 | . | . | . | . | . | . | . | . | . | 68 | . | . | . | . | . | . | . | . | . | . | . | . | . | . | . | . | . | . |
| Hal.str | 4 | 1 | . | . | . | . | . | . | . | . | . | . | . | . | . | . | 5 | . | . | 27 | . | . | . | . | . | . | . | . | . | . | . | . | . | 72 | . | . | . | . | . | . | . | . | . | 60 | . | 55 | . | . |
| Hal.sul | . | . | . | . | . | . | . | . | . | . | . | . | . | . | . | . | . | . | . | . | . | . | . | . | . | . | . | . | . | . | . | . | . | . | . | . | . | . | . | . | 11.4 | . | . | . | . | . | . | . |
| Hal.ver | . | 6 | . | . | . | 15 | . | 27 | 17.7 | 1.5 | . | . | . | . | . | . | . | . | 7.5 | 12.5 | . | . | . | . | . | . | . | . | . | . | . | . | . | . | . | . | . | . | . | . | 50 | . | . | . | . | . | . | . |
| Hel.bac | . | . | . | . | . | . | . | . | . | . | . | . | . | . | . | . | . | . | . | . | . | . | . | . | . | . | . | . | . | . | . | . | . | . | . | 20 | . | . | . | . | . | . | 0.9 | . | . | . | . | . |
| Hel.sal | . | . | . | . | . | 1 | 4 | . | . | . | 7 | . | . | . | 1 | . | . | . | . | . | . | . | . | . | . | . | . | . | . | . | . | . | . | . | . | . | . | . | . | . | . | . | . | . | . | . | . | . |
| Hip.bis | . | . | . | . | . | . | . | . | . | . | . | . | . | . | . | . | . | . | . | . | . | . | . | . | . | . | . | . | . | . | . | . | . | . | . | . | 50 | . | . | . | . | . | . | . | . | . | . | . |
| Hol.glu | . | . | . | . | . | 2 | . | . | . | . | . | . | . | . | . | . | . | . | . | . | . | . | . | . | . | . | . | 1 | . | . | . | . | . | . | . | . | . | . | . | . | . | . | . | . | . | . | . | . |
| Hol.umb | . | . | . | . | . | 1.62 | . | . | . | . | . | . | . | . | 2 | . | . | . | . | . | . | . | . | . | 2 | 0.1 | . | . | . | 4 | . | . | . | . | . | . | . | . | . | . | . | . | . | . | . | . | . | . |
| Hor.mur | . | . | . | . | . | . | 17.8 | 4.5 | 25 | 25 | . | . | . | . | 20 | . | . | . | . | . | . | . | . | 0.5 | . | . | . | . | . | 27 | . | . | . | . | . | . | . | . | . | . | . | . | . | . | . | . | . | . |
| Hor.pro | . | . | . | . | . | . | 0.75 | . | . | . | . | . | . | . | . | . | . | . | . | . | . | . | . | . | . | . | . | . | . | 5 | . | . | . | . | . | . | . | . | . | . | . | . | . | . | . | . | . | . |
| Hyp.pen | . | . | . | . | . | . | . | . | . | 0.26 | . | . | . | . | . | . | . | . | . | . | . | . | . | . | 10.7 | . | . | . | . | . | . | . | . | . | . | . | . | . | . | . | . | . | . | . | . | . | . | . |
| Inu.auc | . | . | . | 2.4 | . | . | . | . | . | . | . | . | . | . | . | . | . | . | . | . | . | . | . | . | . | . | . | . | . | . | . | . | . | . | . | . | . | . | . | . | . | . | . | . | . | . | . | . |
| Iri.pse | . | . | . | . | . | 2.5 | . | . | . | . | . | . | . | . | . | . | . | . | . | . | . | . | . | . | . | . | . | . | . | . | . | . | . | . | . | . | . | . | . | . | . | . | . | . | . | . | . | . |
| Iri.spu | . | . | . | . | . | 4 | . | . | . | . | . | . | . | . | . | . | . | . | . | . | . | . | . | . | . | . | . | . | . | . | . | . | . | . | . | . | . | . | . | . | . | . | . | . | . | . | . | . |
| Jun.hel | . | 9.5 | 100 | 85.6 | 82 | 75.6 | . | . | . | . | . | . | . | . | . | . | . | . | . | . | . | 65 | . | . | . | . | . | . | . | . | . | . | . | . | . | . | . | . | . | . | . | . | . | . | . | . | . | . |
| Koe.lin | . | . | . | . | . | . | 1.6 | . | . | . | . | . | . | . | . | . | . | . | . | . | . | . | . | . | . | . | . | . | . | . | . | . | . | . | . | . | . | . | . | . | . | . | . | . | . | . | . | . |
| Son.asp | . | . | . | . | . | . | . | . | . | . | . | . | . | . | . | . | . | . | . | . | . | . | . | . | . | . | . | . | . | . | . | . | . | . | . | . | . | . | . | . | . | . | . | . | 0.8 | . | . | . |
| Lam.amp | . | . | . | . | . | 0.65 | . | . | . | . | . | . | . | . | . | . | . | . | . | . | . | . | . | . | 1 | . | . | . | . | . | . | . | . | . | . | . | . | . | . | . | . | . | . | . | . | . | . | . |
| Lap.sp. | . | . | . | . | . | . | . | . | . | . | . | . | . | . | . | . | . | . | . | . | . | . | . | . | 1 | . | . | . | . | . | . | . | . | . | . | . | . | . | . | . | . | . | . | . | . | . | . | . |
| Lap.spi | . | . | . | . | . | . | . | . | . | . | . | . | . | . | 0.5 | . | . | . | . | . | . | . | . | . | . | . | . | . | . | . | . | . | . | . | . | . | . | . | . | . | . | . | . | . | . | . | . | . |
| Las.mus | . | . | . | . | . | . | . | . | . | . | . | . | . | . | . | . | . | . | . | . | . | . | . | . | . | . | . | . | . | . | . | . | . | . | . | . | . | . | . | . | . | . | . | . | 0.7 | . | . | . |
| Lau.muc | . | . | . | . | . | . | . | . | . | . | . | . | . | . | . | . | . | . | . | . | . | . | . | . | . | . | . | . | . | . | . | . | . | . | . | 3 | . | . | . | . | . | . | . | . | . | . | . | . |
| Lau.pro | . | . | . | . | . | . | . | . | . | . | . | . | . | . | . | . | . | . | . | . | . | . | . | . | . | . | . | . | . | . | . | . | . | . | 0.8 | . | . | . | . | . | . | . | . | . | . | . | . | . |
| Lep.car | . | 5 | . | . | . | 10.25 | . | . | . | . | . | . | . | . | . | . | . | . | . | . | . | . | . | . | . | . | . | . | . | . | . | . | . | . | . | . | . | . | . | . | . | . | . | . | . | . | . | . |
| Lep.dra | . | . | . | . | . | . | . | . | . | 1 | . | . | . | . | . | . | . | . | . | . | . | . | . | . | . | . | . | . | . | . | . | . | . | . | . | . | . | . | . | . | . | . | . | . | . | . | . | . |
| Lep.dra | . | . | . | 4 | . | 5.2 | . | . | 0.62 | . | . | . | . | . | . | . | . | . | . | . | . | . | . | . | . | 0.5 | . | . | . | . | . | . | . | . | . | . | . | . | . | . | . | . | . | . | . | . | . | . |
| Lep.fil | . | . | . | . | . | . | . | . | . | 0.5 | . | . | . | . | . | . | . | . | . | . | . | . | . | . | . | . | . | . | . | . | . | . | . | . | . | . | . | . | . | . | . | . | . | . | . | . | . | . |
| Lep.per | 0.5 | . | . | . | . | . | . | . | . | . | . | . | . | . | . | . | . | . | . | . | . | . | . | . | . | . | 0.5 | . | . | . | . | . | . | . | . | . | . | . | . | . | . | . | . | . | . | . | 0.7 | . |
| Lep.ves | . | . | . | . | . | . | . | . | . | . | . | . | . | . | . | . | . | . | . | . | . | . | . | 15 | . | . | . | . | . | . | . | . | . | . | . | . | . | . | . | . | . | . | . | . | . | . | . | . |
| Lim.axi | . | . | . | . | . | . | . | . | . | . | . | . | . | . | . | . | . | . | . | . | . | . | . | . | . | . | . | . | . | . | . | . | . | . | . | . | . | 25 | . | . | . | . | . | . | . | . | . | . |
| Lim.fai | . | . | . | . | . | . | . | . | . | . | . | . | . | . | . | . | . | . | . | . | . | . | . | . | . | . | . | . | . | . | . | . | . | . | . | . | . | 49 | . | . | . | . | . | . | . | 1.4 | . | . |
| Lim.mey | . | 0.1 | . | . | . | 0.5 | . | . | . | . | . | . | . | . | . | . | . | . | . | . | . | . | . | . | . | . | . | . | . | . | . | . | . | . | . | . | . | . | . | . | . | . | . | . | . | . | . | . |
| Lin.sim | . | . | . | . | . | . | . | . | . | . | . | . | . | . | . | . | . | . | . | . | . | . | . | . | . | . | . | . | . | 0.5 | . | . | . | . | . | . | . | . | . | . | . | . | . | . | . | . | . | . |
| Lit.arv | . | . | . | . | . | . | . | . | . | . | . | . | . | . | 0.5 | . | . | . | . | . | . | . | . | . | . | . | . | . | . | . | . | . | . | . | . | . | . | . | . | . | . | . | . | . | . | . | . | . |
| Lot.hal | . | . | . | . | . | . | . | . | . | . | . | . | . | . | . | . | . | . | . | . | . | . | . | . | . | . | . | . | . | . | . | . | . | . | 3 | . | . | . | . | . | . | . | . | . | . | . | . | . |
| Lyc.rut | . | . | . | . | . | . | 34 | . | . | . | . | . | . | . | . | . | . | . | . | . | . | . | . | . | . | . | . | . | . | . | . | . | . | . | . | . | . | . | . | . | . | . | . | . | . | . | . | . |
| Lyc.sha | . | . | . | . | . | . | . | . | . | . | . | . | . | . | . | . | . | . | . | . | . | . | . | . | . | . | . | . | . | . | . | . | . | . | . | . | . | . | . | . | . | 72 | . | . | . | . | . | . |
| Mal.afr | . | . | . | . | . | . | . | . | 0.5 | . | . | . | . | . | . | . | . | . | . | . | . | . | . | . | . | . | . | . | . | 0.8 | . | . | . | . | . | . | . | . | . | . | . | . | . | . | . | . | . | . |
| Mal.par | . | . | . | . | . | . | . | . | . | . | . | . | . | . | . | . | . | . | . | . | . | . | . | . | . | . | . | . | . | . | . | . | . | . | . | . | . | . | . | . | . | . | 0.9 | . | . | . | . | . |
| Mal.str | . | . | . | . | . | . | . | . | . | 0.5 | . | . | . | . | . | . | . | . | . | . | . | . | . | . | . | . | . | . | . | . | . | . | . | . | . | . | . | . | . | . | . | . | . | . | . | . | . | . |
| Med.pol | . | . | . | . | . | . | . | . | . | . | . | . | . | . | . | . | . | . | . | . | . | . | . | . | . | . | . | . | . | . | . | . | . | . | . | . | . | . | . | . | 1.8 | . | . | . | . | . | . | . |
| Mes.nod | . | . | . | . | . | . | . | . | . | . | . | . | . | . | . | . | . | . | . | . | . | . | . | . | . | . | . | . | . | . | . | . | . | . | . | . | . | . | . | . | . | . | 35 | 2.4 | . | . | . | . |
| Neo.tor | . | . | . | . | . | . | . | 3 | . | . | 0.75 | . | . | . | . | . | . | . | . | . | . | . | . | . | . | . | . | . | . | . | . | . | . | . | . | . | . | . | . | . | . | . | . | . | . | . | . | . |
| Nit.sch | . | 6 | . | . | . | . | . | . | . | . | . | . | . | . | . | . | . | . | . | . | . | . | . | . | . | . | . | . | . | . | . | . | . | . | . | . | . | . | . | . | . | . | . | . | . | . | 22 | . |
| Non.cas | . | . | . | . | . | . | . | . | . | . | . | . | . | . | . | . | . | . | . | . | . | . | . | . | 0.5 | . | . | . | . | . | . | . | . | . | . | . | . | . | . | . | . | . | . | . | . | . | . | . |
| Och.bac | . | . | . | . | . | . | . | . | . | . | . | . | . | . | . | . | . | . | . | . | . | . | . | . | . | . | . | . | . | . | . | . | . | . | . | . | . | . | . | . | . | . | 3.4 | . | . | . | . | . |
| Oli.pum | . | . | . | . | . | 1 | . | . | 9.5 | . | . | . | . | . | . | . | . | . | . | . | . | . | . | 0.5 | . | . | 30 | . | . | 0.5 | . | . | . | . | . | . | . | . | . | . | . | . | . | . | . | . | . | . |
| Pap.arg | . | . | . | . | . | . | . | . | . | . | . | . | . | . | 0.5 | . | . | . | . | . | . | . | . | . | . | . | . | . | . | . | . | . | . | . | . | . | . | . | . | . | . | . | . | . | . | . | . | . |
| Par.als | . | . | . | . | . | . | . | . | . | . | . | . | . | . | . | . | . | . | . | . | . | . | . | . | . | . | . | . | . | . | . | . | . | . | . | . | . | . | . | . | . | . | 0.5 | . | . | . | . | . |
| Peg.har | . | . | . | . | . | . | 3.5 | . | . | 0.75 | . | . | . | . | 3 | . | . | . | . | . | . | . | . | . | . | . | . | . | . | . | . | . | . | . | . | . | . | . | . | . | . | . | . | . | . | . | . | . |
| Pet.bra | 0.7 | . | . | . | . | . | . | . | . | . | . | . | . | . | . | . | . | . | . | . | . | . | . | . | . | . | . | . | . | . | . | . | . | . | . | . | . | . | . | . | . | . | . | . | . | . | . | . |
| Pet.gla | . | . | . | . | . | . | . | . | . | . | . | . | . | . | . | . | . | . | . | . | . | . | . | . | . | . | . | . | . | . | . | . | . | . | . | . | . | . | . | . | . | . | . | . | . | . | . | 1.6 |
| Phr.aus | . | 3 | . | 9.2 | . | 5.87 | . | . | . | . | . | . | . | . | . | . | . | . | . | . | . | . | . | . | . | . | . | . | . | . | . | . | . | 94 | . | . | . | . | . | . | . | . | . | . | . | . | . | . |
| Pla.amp | . | . | . | . | . | . | . | . | . | . | . | . | . | . | . | . | . | . | . | . | . | . | . | . | . | . | . | . | . | . | . | . | . | . | . | . | . | . | . | . | 0.5 | . | . | . | . | . | . | . |
| Pla.boi | . | . | . | . | . | . | . | . | . | . | . | . | . | . | . | . | . | . | . | . | . | . | . | . | . | . | . | . | . | . | . | . | . | . | . | 15 | . | . | . | . | . | . | . | . | . | . | . | . |
| Pla.cor | . | . | . | . | . | . | . | . | . | . | . | . | . | . | . | . | . | . | . | . | . | . | . | . | . | . | . | . | . | . | . | . | . | . | . | . | . | . | . | . | . | . | . | 5 | . | . | . | . |
| Pla.mar | . | . | . | . | . | . | . | 1 | . | . | . | . | . | . | . | . | . | . | . | . | . | . | . | . | . | . | . | . | . | . | . | . | . | . | . | . | . | . | . | . | . | . | . | . | . | . | . | . |
| Poa.bul | . | . | . | . | . | . | . | . | . | 31.6 | . | . | . | . | . | . | . | . | . | . | . | . | . | . | 1 | . | . | . | . | . | . | . | . | . | . | . | . | . | . | . | . | . | . | . | . | . | . | . |
| Pol.pat | . | . | . | . | . | . | . | . | . | . | . | . | . | . | . | . | . | . | . | . | . | . | . | . | . | . | . | 1 | . | 1.6 | . | . | . | . | . | . | . | . | . | . | . | . | . | . | . | . | . | . |
| Psy.spi | . | . | . | . | . | . | . | . | . | . | . | . | . | . | . | . | . | . | . | . | . | . | . | . | . | . | . | . | . | . | . | . | . | . | . | . | . | . | . | . | . | . | . | 24 | . | . | . | . |
| Puc.bul | . | . | . | . | . | 4.6 | . | . | . | . | . | . | . | . | . | . | . | . | . | . | . | . | . | . | . | . | . | . | . | . | . | . | . | . | . | . | . | . | . | . | . | . | . | . | . | . | . | . |
| Puc.dis | . | . | . | . | . | . | . | . | . | . | . | . | . | . | . | . | . | . | . | . | . | . | . | . | . | . | . | . | . | . | . | . | . | . | . | . | . | . | . | . | . | . | . | . | . | . | 5.2 | . |
| Pul.ara | . | . | . | . | . | . | . | . | . | . | . | . | . | . | . | . | . | . | . | . | . | . | . | . | . | . | . | . | . | . | . | . | . | . | . | . | . | . | . | . | . | . | 0.5 | . | . | . | . | . |
| Rea.alt | . | . | . | . | . | . | . | . | . | . | . | . | . | . | . | . | . | . | . | . | . | . | . | . | . | . | . | . | . | . | . | . | . | . | . | . | . | . | . | . | . | . | . | . | . | . | 0.5 | . |
| Rei.ori | . | . | . | . | . | . | . | . | . | . | . | . | . | . | . | . | . | . | . | . | . | . | . | . | . | . | . | . | . | . | . | . | . | . | . | . | . | . | . | . | . | . | . | . | 0.5 | . | . | . |
| Roc.dis | . | . | . | . | . | . | . | . | . | . | . | . | . | . | 0.5 | . | . | . | . | . | . | . | . | . | . | . | . | . | . | . | . | . | . | . | . | . | . | . | . | . | . | . | . | . | . | . | . | . |
| Roe.hyb | . | . | . | . | . | . | . | . | . | 0.5 | . | . | . | . | 0.5 | . | . | . | . | . | . | . | . | . | 0.5 | . | . | . | . | 0.5 | . | . | . | . | . | . | . | . | . | . | . | . | . | . | . | . | . | . |
| Rum.ves | . | . | . | . | . | . | . | . | . | . | . | . | . | . | . | . | . | . | . | . | . | . | . | . | . | . | . | . | . | . | . | . | . | . | . | . | . | . | . | . | . | . | . | . | 0.5 | . | . | . |
| Sal.ira | . | . | . | . | . | . | . | . | . | . | . | . | . | . | . | . | 17.5 | . | . | . | 26 | . | . | . | . | . | . | . | . | 5 | . | 64 | . | . | . | . | . | . | . | . | . | . | . | . | . | . | . | . |
| Sal.sin | . | . | . | . | . | . | . | . | . | . | . | . | . | . | . | . | . | . | . | . | . | . | . | . | . | . | . | . | . | . | . | . | . | . | . | . | . | . | 2 | . | . | . | . | . | . | 7.8 | . | . |
| Sau.sal | . | . | . | 2.5 | 10.6 | . | . | . | . | . | . | . | . | . | . | . | . | . | . | . | . | . | . | . | . | . | . | . | . | . | . | . | . | . | . | . | . | . | . | . | . | . | . | . | . | . | . | . |
| Scl.dur | 0.5 | . | . | . | . | 1 | . | . | . | 0.5 | . | . | . | . | . | . | . | . | . | . | . | . | . | . | . | . | 0.5 | . | . | . | . | . | . | . | . | . | . | . | . | . | . | . | . | . | . | . | . | . |
| Sco.lac | . | . | . | . | . | 2.5 | . | 0.53 | 1.1 | . | . | . | . | . | . | . | . | . | . | . | . | . | . | . | . | . | . | . | . | . | . | . | . | . | . | . | . | . | . | . | . | . | . | . | . | . | . | . |
| Sco.mur | . | . | . | . | . | . | . | . | . | . | . | . | . | . | . | . | . | . | . | . | . | . | . | . | . | . | . | . | . | . | . | . | . | . | . | . | . | . | . | . | 8.6 | . | . | . | . | . | . | . |
| Sen.gla | . | . | . | . | . | 2.52 | 3 | . | 0.5 | 2.42 | 2 | . | . | . | 0.5 | . | . | . | . | . | . | . | . | . | . | . | . | . | . | 20 | . | . | . | . | . | 10.6 | . | . | . | . | . | . | . | . | . | . | . | . |
| Sen.ver | . | . | . | . | . | . | . | . | . | . | . | . | . | . | . | . | . | . | . | . | . | . | . | 0.5 | . | . | 2.5 | . | . | . | . | . | . | . | . | . | . | . | . | . | . | . | . | . | . | . | . | . |
| Sil.cni | . | . | . | . | . | 0.65 | 0.5 | 0.1 | . | . | . | . | . | . | 0.5 | . | . | . | . | . | . | . | . | . | . | . | . | . | . | . | . | . | . | . | . | . | . | . | . | . | . | . | . | . | . | . | . | . |
| Sil.con | . | . | . | . | . | . | . | . | . | . | . | . | . | . | 0.5 | . | . | . | . | . | . | . | . | . | . | . | . | . | . | . | . | . | . | . | . | . | . | . | . | . | . | . | . | . | . | . | . | . |
| Sis.sep | . | . | . | . | . | . | 23.5 | . | 0.4 | 0.5 | 6 | . | . | . | . | . | . | . | . | . | . | . | . | 0.5 | . | . | . | . | . | . | . | . | . | . | . | . | . | . | . | . | . | . | . | . | . | . | . | . |
| Sod.dru | . | . | . | . | . | . | . | . | . | . | . | . | . | . | . | . | . | . | . | . | . | . | . | . | . | . | . | . | . | . | . | . | . | . | . | . | . | . | . | . | . | . | . | . | 17 | . | . | . |
| Sod.ine | . | . | . | . | . | . | . | . | . | . | . | . | . | . | . | . | . | . | . | . | . | . | . | . | . | . | . | . | . | . | 92 | . | . | . | . | . | . | . | . | . | . | . | . | . | . | . | . | . |
| Sol.inc | . | . | . | . | . | . | . | . | . | . | . | . | . | . | . | . | . | . | . | . | . | . | . | . | . | . | . | . | . | . | . | . | . | . | . | . | . | . | . | . | . | . | 0.5 | . | . | . | . | . |
| Spe.dia | . | . | . | . | . | . | . | . | . | . | . | . | . | . | 0.5 | . | . | . | . | . | . | . | . | . | . | . | . | . | . | . | . | . | . | . | . | . | . | . | . | . | . | . | . | 7.8 | . | . | . | . |
| Spe.mar | . | . | . | . | . | . | . | . | . | . | . | . | . | . | . | . | . | . | . | . | . | . | . | . | . | . | . | . | . | . | . | . | . | 0.7 | . | . | . | . | . | . | . | . | . | . | . | . | . | . |
| Spi.tet | . | . | . | . | . | . | . | . | 0.6 | 3.5 | . | . | . | . | . | . | . | . | . | . | . | . | . | . | . | . | . | . | . | . | . | . | . | . | . | . | . | . | . | . | . | . | . | . | . | . | . | . |
| Spo.ara | . | . | . | . | . | . | . | . | . | . | . | . | . | . | . | . | . | . | . | . | . | . | . | . | . | . | . | . | . | . | . | . | . | . | . | . | . | 47 | . | . | . | . | . | . | . | . | . | . |
| Sti.cap | . | . | . | . | . | . | . | . | . | . | . | . | . | . | . | . | . | . | . | . | . | . | . | . | . | . | . | . | . | . | . | . | . | . | . | . | . | . | . | . | . | . | 0.8 | . | . | . | . | . |
| Sti.hoh | . | . | . | . | . | . | . | . | . | . | 0.75 | . | . | . | . | . | . | . | . | . | . | . | . | . | . | . | . | . | . | . | . | . | . | . | . | . | . | . | . | . | . | . | . | . | . | . | 19 | . |
| Sua.acu | . | . | . | . | . | . | . | . | . | . | . | . | . | . | . | 30 | 21 | . | . | . | . | . | . | . | . | . | . | . | . | 5 | . | . | . | . | . | . | . | . | . | . | . | . | . | . | . | . | . | . |
| Sua.aeg | . | . | . | . | . | . | . | . | . | . | . | . | . | . | . | . | . | . | . | . | . | . | . | . | . | . | . | . | . | . | . | . | . | . | . | . | . | . | . | . | . | . | . | 0.5 | . | . | . | . |
| Sua.alt | 1.2 | . | . | . | . | . | . | . | . | . | . | 0.7 | 50 | 68 | 17.2 | 0.5 | . | . | . | . | . | . | . | . | . | 0.5 | . | 32 | . | 2.6 | . | . | . | . | . | . | . | . | . | . | . | . | . | . | . | . | . | . |
| Sua.fru | . | . | . | . | . | . | . | . | . | . | . | . | . | . | . | . | . | . | . | . | . | . | . | . | . | . | . | . | . | . | . | . | . | . | . | . | . | 0.7 | . | . | . | . | 1.4 | . | . | 5.8 | . | . |
| Sua.ira | . | . | . | . | . | . | . | . | . | . | . | . | . | . | . | . | . | . | . | . | . | . | . | . | . | . | . | . | . | . | . | . | . | . | . | . | . | . | . | . | . | . | . | . | . | 4.6 | . | . |
| Sua.kha | . | . | . | . | . | . | . | . | . | . | . | . | . | . | . | . | . | . | . | . | . | . | . | . | . | . | . | . | . | . | . | . | . | . | . | . | . | . | . | . | . | . | . | . | . | 8.8 | . | . |
| Tam.and | . | . | . | . | . | . | . | . | . | . | . | . | . | . | . | . | . | . | . | . | . | . | . | . | . | . | . | . | . | 20 | . | . | . | . | . | . | . | . | . | . | . | . | . | . | . | . | . | . |
| Tam.oct | . | . | . | . | . | . | . | . | . | . | . | . | . | . | . | . | . | . | . | . | . | . | . | . | . | . | . | . | 80 | . | . | . | . | . | . | . | . | . | . | . | . | . | . | . | . | . | . | . |
| Tam.pyc | . | . | . | . | . | . | . | . | . | . | . | . | . | . | . | . | . | . | . | . | . | . | . | . | . | . | . | . | . | . | . | . | . | . | . | . | . | . | . | . | . | . | . | 4 | . | . | . | . |
| Tar.sp.1 | . | . | . | 0.5 | . | . | . | . | . | . | . | . | . | . | . | . | . | . | . | . | . | . | . | . | . | . | . | . | . | . | . | . | . | . | . | . | . | . | . | . | . | . | . | . | . | . | . | . |
| Tar.sp.2 | . | . | . | . | . | 2 | . | . | . | . | . | . | . | . | . | . | . | . | . | . | . | . | . | . | . | . | . | . | . | . | . | . | . | . | . | . | . | . | . | . | . | . | . | . | . | . | . | . |
| Tet.ten | . | . | . | . | . | . | . | . | . | 6.4 | . | . | . | . | . | . | . | . | . | . | . | . | . | . | . | . | . | . | . | . | . | . | . | . | . | . | . | . | . | . | . | . | . | . | . | . | . | . |
| The.com | . | . | . | . | . | 0.5 | . | . | . | . | . | . | . | . | . | . | . | . | . | . | . | . | . | . | . | . | . | . | . | . | . | . | . | . | . | . | . | . | . | . | . | . | . | . | . | . | . | . |
| Tra.gra | . | . | . | . | . | 1 | . | . | . | . | . | . | . | . | . | . | . | . | . | . | . | . | . | . | . | . | . | . | . | . | . | . | . | . | . | . | . | . | . | . | . | . | . | . | . | . | . | . |
| Tri.arc | . | . | . | . | . | . | 1 | . | 0.5 | 0.75 | . | . | . | . | . | . | . | . | . | . | . | . | . | . | . | . | . | . | . | . | . | . | . | . | . | . | . | . | . | . | . | . | . | . | . | . | . | . |
| Tri.coe | . | . | . | . | . | . | . | . | . | . | . | . | . | . | 0.5 | . | . | . | . | . | . | . | . | . | . | . | . | . | . | . | . | . | . | . | . | . | . | . | . | . | . | . | . | . | . | . | . | . |
| Tri.par | . | . | . | . | . | 0.82 | 5.8 | 8.875 | 1.5 | 29.3 | . | . | . | . | 1 | . | . | . | . | . | . | . | . | . | . | . | 0.5 | . | . | . | . | . | . | . | . | . | . | . | . | . | . | . | . | . | . | . | . | . |
| Typ.gro | . | . | . | . | . | . | . | . | . | . | . | . | . | . | . | . | . | . | . | . | . | 65 | . | . | . | . | . | . | . | . | . | . | . | . | . | . | . | . | . | . | . | . | . | . | . | . | . | . |
| Val.pla | . | . | . | . | . | . | . | . | . | . | . | . | . | . | . | . | . | . | . | . | . | . | . | . | 0.5 | . | . | . | . | . | . | . | . | . | . | . | . | . | . | . | . | . | . | . | . | . | . | . |
| Ver.cam | . | . | . | . | . | 0.26 | . | . | . | 1.25 | . | . | . | . | . | . | . | . | . | . | . | . | . | . | 0.5 | . | . | . | . | . | . | . | . | . | . | . | . | . | . | . | . | . | . | . | . | . | . | . |
| Ver.per | . | . | . | . | . | . | . | . | . | . | . | . | . | . | . | . | . | . | . | . | . | . | . | . | . | . | . | . | 0.5 | . | . | . | . | . | . | . | . | . | . | . | . | . | . | . | . | . | . | . |
| Vio.occ | . | . | . | . | . | . | . | . | . | . | . | . | . | . | . | . | . | . | . | . | . | . | . | . | . | . | . | . | 0.6 | . | . | . | . | . | . | . | . | . | . | . | . | . | . | . | . | . | . | . |
| Vul.per | . | . | . | . | . | . | . | . | 2 | . | . | . | . | . | . | . | . | . | . | . | . | . | . | . | . | . | . | . | . | . | . | . | . | . | . | . | . | . | . | . | . | . | . | . | . | . | . | . |
| Ziz.num | . | . | . | . | . | . | . | . | . | . | . | . | . | . | . | . | . | . | . | . | . | . | . | . | . | . | . | . | . | . | . | . | . | . | . | . | . | . | . | . | . | . | 12 | . | . | . | . | . |
| Zyg.fab | . | . | . | . | . | 0.5 | . | . | . | . | . | . | . | . | . | . | . | . | . | . | . | . | . | . | . | 18.7 | . | . | . | . | . | . | . | . | . | . | . | . | . | . | . | . | . | . | . | . | . | . |

Supporting Information to the paper Matinzadeh, Z. Functional structure of plant communities along salinity gradients in Iranian salt marshes. *Plant-Environment Interactions*.

Appendix S4. Mean soil variables of five samples in 48 studied plots. Electrical Conductivity (EC; dS/m) and concentrations of some key elements (mg g^−1^) are shown.

| **Plot/Soil variables** | **pH** | **EC** | **Carbonate** | **Gypsum** | **OM** | **Al** | **As** | **Ca** | **Co** | **Cr** | **Cu** | **Fe** | **K** | **Li** | **Mg** | **Mn** | **Na** | **Ni** | **P** | **Pb** | **S** | **Si** | **Ti** | **V** | **Zn** | **N** | **Ct** | **Sand** | **Silt** | **Clay** |
| --- | --- | --- | --- | --- | --- | --- | --- | --- | --- | --- | --- | --- | --- | --- | --- | --- | --- | --- | --- | --- | --- | --- | --- | --- | --- | --- | --- | --- | --- | --- |
| **U11** | 9 | 12.74 | 636.9 | 293.6 | 13.6 | 10.7 | 0.2 | 278 | 0 | 0 | 0 | 8.76 | 3.31 | 0 | 18.7 | 0.6 | 20.5 | 0 | 0.4 | 0 | 5.03 | 4.6 | 0.4 | 0 | 0 | 18.68 | 96.16 | 741.5 | 225.84 | 32.66 |
| **U12** | 9.3 | 17.38 | 406.4 | 728.6 | 34.8 | 20.9 | 0.2 | 145.4 | 0 | 0 | 0 | 14.1 | 6.67 | 0.1 | 49.4 | 0.4 | 28.6 | 0 | 0.6 | 0.01 | 12.1 | 5 | 0.8 | 0 | 0.1 | 3.02 | 77.06 | 355.8 | 570.66 | 73.5 |
| **U13** | 8.8 | 0.53 | 531.6 | 633.2 | 50 | 21.6 | 0.1 | 200.2 | 0 | 0 | 0 | 14.5 | 5.72 | 0.1 | 40 | 0.6 | 1.68 | 0 | 0.6 | 0.01 | 1.47 | 5.1 | 0.8 | 0 | 0.1 | 17.04 | 103.2 | 610.9 | 345.08 | 43.98 |
| **U14** | 8.7 | 0.62 | 495.6 | 703.6 | 60.9 | 20.5 | 0.2 | 190.6 | 0 | 0 | 0 | 14.1 | 5.63 | 0.1 | 40.3 | 0.6 | 1.69 | 0 | 0.8 | 0.01 | 1.77 | 4.7 | 0.7 | 0 | 0.1 | 22.25 | 105.5 | 607.9 | 347.88 | 44.22 |
| **U15** | 8.9 | 0.40 | 334.1 | 694.8 | 58 | 34.5 | 0.3 | 122 | 0 | 0.1 | 0 | 21 | 8.78 | 0.1 | 48.9 | 0.7 | 1.11 | 0.1 | 0.9 | 0.01 | 0.92 | 4.3 | 1.2 | 0.1 | 0.1 | 20.13 | 86.11 | 543.7 | 399.4 | 56.86 |
| **U16** | 8.5 | 0.57 | 297 | 725 | 45.9 | 39.4 | 0.2 | 132.9 | 0 | 0.1 | 0 | 27.7 | 9.69 | 0 | 25 | 0.7 | 0.81 | 0.1 | 0.9 | 0.01 | 1.17 | 4.7 | 1.4 | 0.1 | 0.1 | 31.21 | 70.88 | 402.6 | 525.08 | 72.36 |
| **U17** | 8.4 | 0.16 | 388.6 | 434.5 | 27 | 29.2 | 0.1 | 172.7 | 0 | 0.1 | 0 | 23.5 | 6.86 | 0 | 13.5 | 0.8 | 0.81 | 0 | 0.8 | 0.01 | 0.56 | 4.9 | 1.5 | 0.1 | 0.1 | 1.82 | 72.91 | 582.6 | 376.36 | 41.06 |
| **U18** | 8.8 | 0.51 | 286.1 | 624.2 | 49.5 | 37.4 | 0.1 | 125.7 | 0 | 0.1 | 0 | 26.6 | 10.7 | 0.1 | 21.4 | 0.7 | 1.49 | 0.1 | 1 | 0.01 | 0.78 | 4.6 | 1.5 | 0.1 | 0.1 | 3.392 | 71.87 | 472.4 | 476.6 | 51.02 |
| **U19** | 8.5 | 0.37 | 292.5 | 520 | 51.5 | 39.6 | 0.1 | 133.3 | 0 | 0.1 | 0 | 27.5 | 10.2 | 0 | 17.6 | 0.7 | 0.98 | 0.1 | 1 | 0.01 | 0.87 | 4.8 | 1.5 | 0.1 | 0.1 | 27.7 | 74.93 | 464.3 | 482.42 | 53.3 |
| **U110** | 8.7 | 0.21 | 344 | 631 | 22.6 | 36.2 | 0.1 | 154.5 | 0 | 0.1 | 0 | 26.6 | 8.35 | 0 | 14.6 | 0.7 | 1.05 | 0.1 | 0.8 | 0.01 | 0.55 | 4.5 | 1.8 | 0.1 | 0.1 | 1.552 | 63.02 | 418.7 | 529.88 | 51.38 |
| **U111** | 8.5 | 0.16 | 449 | 245.6 | 15.5 | 26.2 | 0.3 | 198.8 | 0 | 0 | 0 | 20.1 | 5.69 | 0 | 10.5 | 0.6 | 0.47 | 0 | 0.6 | 0.01 | 0.39 | 5.5 | 1.4 | 0.1 | 0.1 | 1.175 | 72.48 | 636.9 | 324.4 | 38.65 |
| **U21** | 8.3 | 7.25 | 149.3 | 656.6 | 12.3 | 52.6 | 0 | 67.32 | 0 | 0.1 | 0.1 | 37.1 | 29.6 | 0 | 35.4 | 0.9 | 10.4 | 0.1 | 1.3 | 0.02 | 9.46 | 4 | 1.5 | 0.1 | 0.1 | 5.928 | 25.37 | 45.78 | 804.6 | 149.6 |
| **U22** | 8.3 | 7.00 | 152 | 823.3 | 21.6 | 54 | 0 | 72.54 | 0 | 0.1 | 0.1 | 38.1 | 22.5 | 0.1 | 30.4 | 0.9 | 10.6 | 0.1 | 1.5 | 0.02 | 7.84 | 5.1 | 1.7 | 0.1 | 0.1 | 2.334 | 32.76 | 216.7 | 665.64 | 117.7 |
| **U23** | 8.5 | 9.85 | 72.33 | 609.5 | 21.2 | 43.6 | 0 | 41.63 | 0 | 0.1 | 0.1 | 36.7 | 26 | 0 | 22.1 | 0.6 | 24.8 | 0.1 | 4.2 | 0.02 | 7.04 | 5.6 | 2.4 | 0.1 | 0.1 | 1.634 | 22.19 | 400.3 | 524.76 | 74.92 |
| **U24** | 9.2 | 3.42 | 54.35 | 575.9 | 13.3 | 35 | 0 | 35.29 | 0 | 0.1 | 0.1 | 36.8 | 25.9 | 0 | 15.9 | 0.8 | 14.7 | 0 | 5.5 | 0.02 | 4.45 | 5.3 | 3.1 | 0.1 | 0.1 | 5.616 | 13.42 | 738.2 | 237.04 | 24.82 |
| **U31** | 8.4 | 18.94 | 67.58 | 717.6 | 16.7 | 44.3 | 0 | 31.52 | 0 | 0.1 | 0 | 31 | 16 | 0.1 | 34.2 | 0.8 | 29.7 | 0.1 | 1.2 | 0.02 | 8.13 | 4.8 | 1.7 | 0.1 | 0.1 | 1.308 | 15.12 | 145.1 | 755.78 | 97.75 |
| **U32** | 8.6 | 7.51 | 35.47 | 547.4 | 5.71 | 23.2 | 0 | 14.6 | 0 | 0 | 0 | 24 | 8.14 | 0 | 16.5 | 0.6 | 11.1 | 0 | 1.4 | 0.01 | 3.33 | 5.4 | 1.8 | 0.1 | 0.1 | 0.61 | 5.345 | 428.4 | 520.4 | 51.25 |
| **U33** | 8.8 | 7.16 | 52.77 | 455.3 | 9.36 | 25.4 | 0 | 19.77 | 0 | 0 | 0 | 25.9 | 13 | 0 | 23.7 | 0.6 | 10.9 | 0.1 | 2.1 | 0.02 | 2.28 | 5.9 | 1.7 | 0.1 | 0.1 | 1.06 | 11.13 | 485.7 | 477.36 | 66.67 |
| **U34** | 8.8 | 0.24 | 27.92 | 377.7 | 11.9 | 29.9 | 0 | 14.17 | 0 | 0.1 | 0 | 27.8 | 12.5 | 0 | 26.5 | 0.5 | 1.47 | 0.1 | 2.1 | 0.01 | 0.56 | 3.9 | 1.6 | 0.1 | 0.1 | 0.6 | 9.78 | 605.8 | 345 | 49.2 |
| **U35** | 8.6 | 6.97 | 26.26 | 213.8 | 8.81 | 19.3 | 0 | 13.65 | 0 | 0 | 0 | 22.6 | 6.13 | 0 | 13.5 | 0.4 | 12.7 | 0 | 1.3 | 0.01 | 1.28 | 6 | 2 | 0.1 | 0.1 | 2.798 | 6.04 | 589.6 | 297.7 | 24.76 |
| **U41** | 8.9 | 22.87 | 301.6 | 393.4 | 16.7 | 27.6 | 0 | 129.9 | 0 | 0 | 0 | 23.1 | 8.13 | 0 | 17.8 | 0.7 | 25.2 | 0 | 0.5 | 0.01 | 6.24 | 4.9 | 1.1 | 0.1 | 0.1 | 1.15 | 44.6 | 359.4 | 568.7 | 71.9 |
| **U42** | 8.2 | 4.34 | 317.8 | 479.4 | 28.5 | 29.8 | 0 | 140.4 | 0 | 0.1 | 0 | 23 | 8.08 | 0 | 14.7 | 0.7 | 4.52 | 0 | 0.6 | 0.01 | 5.57 | 5.2 | 1 | 0.1 | 0.1 | 1.978 | 58.73 | 431.7 | 507.76 | 60.52 |
| **U43** | 8 | 1.92 | 202.5 | 403.8 | 7.23 | 41.4 | 0 | 88.06 | 0 | 0.1 | 0 | 30.1 | 10.6 | 0 | 15 | 1.1 | 1.94 | 0.1 | 0.7 | 0.01 | 0.86 | 3.3 | 0.8 | 0.1 | 0.1 | 52.26 | 30.69 | 128.6 | 769.8 | 101.7 |
| **U51** | 8.7 | 1.69 | 671.5 | 179.3 | 6.83 | 13.8 | 0 | 284.1 | 0 | 0 | 0 | 12.3 | 3.12 | 0 | 10.3 | 0.3 | 3.98 | 0 | 0.9 | 0.01 | 9.97 | 5.2 | 0.6 | 0 | 0 | 15.69 | 90.27 | 827.5 | 154.86 | 17.58 |
| **U52** | 8.8 | 0.14 | 558.1 | 150.1 | 12.2 | 23.8 | 0 | 230.6 | 0 | 0.1 | 0 | 19.3 | 5.28 | 0 | 12.4 | 0.4 | 2.12 | 0 | 1.2 | 0.01 | 2.38 | 4.6 | 1 | 0 | 0.1 | 4.934 | 75.35 | 647.4 | 316.94 | 35.58 |
| **U53** | 9.3 | 0.10 | 671.9 | 69.65 | 4.49 | 8.56 | 0.1 | 313.7 | 0 | 0 | 0 | 8.9 | 2.45 | 0 | 8.74 | 0.2 | 2.75 | 0 | 0.8 | 0.01 | 2.82 | 5.6 | 0.4 | 0 | 0 | 32.31 | 100.4 | 948.6 | 47.84 | 3.56 |
| **U54** | 9.5 | 0.43 | 489.1 | 144.5 | 14.4 | 22.5 | 0 | 209.5 | 0 | 0.1 | 0 | 19.8 | 5.39 | 0 | 12.4 | 0.4 | 2.94 | 0 | 1.5 | 0.01 | 1.44 | 4.7 | 1.4 | 0.1 | 0.1 | 61.7 | 73.6 | 653.2 | 317.36 | 28.3 |
| **U55** | 8.9 | 0.23 | 300.7 | 304.8 | 15.9 | 43 | 0 | 137.6 | 0 | 0.1 | 0 | 32.5 | 9.08 | 0 | 15.5 | 0.7 | 1.75 | 0.1 | 1.9 | 0.01 | 1.05 | 3.9 | 1.9 | 0.1 | 0.1 | 66.41 | 49.91 | 414.9 | 517.78 | 67.3 |
| **U56** | 8.5 | 0.52 | 148.7 | 391.3 | 36.8 | 56.7 | 0 | 71.43 | 0 | 0.1 | 0.1 | 42.7 | 17.2 | 0 | 20.2 | 0.9 | 1.56 | 0.1 | 2 | 0.02 | 1.1 | 5.2 | 2.2 | 0.1 | 0.1 | 3.816 | 39.31 | 287.8 | 620.6 | 91.62 |
| **U6** | 9 | 5.86 | 280.3 | 133.7 | 7.78 | 20.7 | 0 | 120.6 | 0 | 0 | 0 | 19.2 | 6.62 | 0 | 12.1 | 0.4 | 8.05 | 0 | 0.5 | 0.01 | 1.42 | 6.1 | 0.9 | 0 | 0 | 1.06 | 37.91 | 821.6 | 158.6 | 19.82 |
| **U7** | 8.3 | 4.88 | 126.9 | 737.1 | 14.8 | 51.4 | 0 | 59.37 | 0 | 0.1 | 0.1 | 37.5 | 25.2 | 0.1 | 37.2 | 0.9 | 8.56 | 0.1 | 1.4 | 0.01 | 2.91 | 5 | 1.4 | 0.1 | 0.1 | 20.71 | 25.72 | 82.9 | 781.02 | 136.1 |
| **U81** | 8.7 | 5.75 | 84.09 | 275.3 | 13.3 | 25.8 | 0 | 41.76 | 0 | 0.2 | 0 | 33.3 | 5.19 | 0 | 25.5 | 0.7 | 6.55 | 0.1 | 0.8 | 0 | 2.34 | 6.5 | 4 | 0.1 | 0.1 | 0.817 | 18.1 | 686.8 | 287.2 | 26.03 |
| **U82** | 8.9 | 18.79 | 93.69 | 287.4 | 12.4 | 33.3 | 0 | 44.07 | 0 | 0.2 | 0 | 39.3 | 9.78 | 0 | 31.3 | 0.8 | 27.5 | 0.2 | 1.2 | 0.01 | 3.18 | 6 | 4.1 | 0.1 | 0.1 | 0.588 | 15.17 | 280.1 | 648.7 | 71.2 |
| **U83** | 8.6 | 14.15 | 123.1 | 395.8 | 16.6 | 28.7 | 0 | 53.26 | 0 | 0.2 | 0 | 33.9 | 8.63 | 0.1 | 34.5 | 0.9 | 19.7 | 0.1 | 1.4 | 0.01 | 1.34 | 4.9 | 4.1 | 0.1 | 0.1 | 0.802 | 22.08 | 288 | 645.2 | 66.84 |
| **N11** | 9.8 | 0.13 | 935 | 22.46 | 1.23 | 0.71 | 0 | 384.2 | 0 | 0 | 0 | 1.09 | 0.23 | 0 | 14.8 | 0 | 2.42 | 0 | 0.9 | 0 | 1.35 | 1.8 | 0 | 0 | 0 | 0.244 | 115.9 | 1000 | 0 | 0 |
| **N12** | 9.6 | 0.90 | 931.2 | 22.03 | 1.99 | 0.88 | 0 | 378.2 | 0 | 0 | 0 | 1.26 | 0.27 | 0 | 14.6 | 0.1 | 2.27 | 0 | 0.9 | 0 | 1.29 | 2.2 | 0 | 0 | 0 | 0.244 | 114.8 | 1000 | 0 | 0 |
| **N13** | 9.3 | 0.10 | 920.8 | 24.12 | 2.52 | 1.4 | 0 | 375.9 | 0 | 0 | 0 | 1.63 | 0.36 | 0 | 14.8 | 0.1 | 2.15 | 0 | 1 | 0 | 1.23 | 3.3 | 0.1 | 0 | 0 | 0.306 | 116.2 | 993.2 | 6.84 | 0 |
| **N14** | 9.7 | 0.14 | 918.6 | 17.84 | 1.74 | 1.49 | 0 | 375.6 | 0 | 0 | 0 | 1.7 | 0.38 | 0 | 15.5 | 0.1 | 2.37 | 0 | 1 | 0 | 1.34 | 3.7 | 0.1 | 0 | 0 | 0.442 | 115.9 | 980.3 | 19.78 | 0 |
| **N15** | 9.3 | 3.21 | 900.4 | 30.38 | 6.14 | 1.36 | 0 | 368.4 | 0 | 0 | 0 | 1.44 | 0.5 | 0 | 14.5 | 0.1 | 4.96 | 0 | 0.9 | 0 | 1.97 | 3.3 | 0 | 0 | 0 | 0.56 | 117.7 | 972.7 | 27.2 | 0.08 |
| **N16** | 9.4 | 2.15 | 903 | 26.33 | 2.9 | 1.82 | 0 | 370.4 | 0 | 0 | 0 | 1.8 | 0.53 | 0 | 16.1 | 0.1 | 4.07 | 0 | 0.9 | 0 | 1.66 | 3.6 | 0.1 | 0 | 0 | 0.268 | 117.2 | 962.6 | 37 | 0.36 |
| **N21** | 8.2 | 1.91 | 449.3 | 308 | 12.3 | 29.5 | 0 | 183.8 | 0 | 0.1 | 0 | 21.7 | 7.63 | 0 | 37.2 | 0.5 | 1.67 | 0.1 | 1.3 | 0.01 | 1.48 | 4.7 | 1.2 | 0.1 | 0.1 | 31.83 | 70.87 | 150.4 | 739.02 | 110.5 |
| **N22** | 8.1 | 1.07 | 430 | 236.2 | 13.9 | 30.2 | 0 | 171.7 | 0 | 0.1 | 0 | 22.3 | 7.5 | 0 | 37.8 | 0.5 | 1.06 | 0.1 | 1.6 | 0.01 | 0.61 | 4.6 | 1.1 | 0.1 | 0.1 | 32.68 | 67.37 | 169.7 | 724.9 | 89.4 |
| **N3** | 8.4 | 6.06 | 458.1 | 460.1 | 17.2 | 10.3 | 0 | 252.9 | 0 | 0 | 0 | 7.96 | 2.7 | 0 | 17.8 | 0.2 | 4.66 | 0 | 5 | 0.03 | 52.1 | 5.2 | 0.5 | 0 | 0 | 74.94 | 74.03 | 545.1 | 298.9 | 30.04 |
| **N4** | 8.3 | 3.43 | 641.7 | 296.3 | 7.59 | 15.1 | 0 | 276.4 | 0 | 0.1 | 0 | 9.73 | 3.28 | 0 | 22.8 | 0.2 | 2.41 | 0 | 0.9 | 0 | 5.06 | 4.4 | 0.7 | 0 | 0 | 0.452 | 98 | 122.3 | 789.28 | 88.42 |
| **N5** | 8.4 | 3.51 | 528.3 | 214.9 | 7.56 | 13.9 | 0 | 242.3 | 0 | 0 | 0 | 10.1 | 3.15 | 0 | 32.8 | 0.3 | 2.65 | 0 | 0.9 | 0 | 1.87 | 4.4 | 0.6 | 0 | 0 | 0.314 | 86.81 | 345.6 | 582.94 | 71.46 |
| **K** | 8.6 | 10.52 | 403.3 | 455.1 | 6.74 | 26.8 | 0 | 178.3 | 0 | 0.1 | 0 | 20.8 | 7.99 | 0 | 50 | 0.5 | 12.9 | 0.1 | 0.8 | 0.01 | 1.72 | 4.7 | 1.3 | 0.1 | 0.1 | 0.322 | 57.57 | 63.89 | 837.375 | 98.37 |
| **M1** | 8.1 | 2.09 | 100.3 | 641.1 | 5.46 | 43.9 | 0 | 75.71 | 0 | 0.1 | 0 | 28.9 | 12 | 0.1 | 61.3 | 0.7 | 0.99 | 0.1 | 1.2 | 0.01 | 32.2 | 3.9 | 0.9 | 0.1 | 0.1 | 109.1 | 17.19 | 621.7 | 290.6 | 41.24 |
| **M2** | 8.3 | 3.61 | 61.48 | 666.7 | 5.73 | 45.7 | 0 | 72.99 | 0 | 0.1 | 0 | 29.4 | 15.1 | 0.1 | 50.7 | 0.7 | 3.98 | 0.1 | 1.1 | 0.01 | 41.9 | 4.3 | 1 | 0.1 | 0.1 | 117.5 | 12.55 | 428.8 | 642.1 | 66.22 |

Supporting Information to the paper Matinzadeh, Z. Functional structure of plant communities along salinity gradients in Iranian salt marshes. *Plant-Environment Interactions*.

Appendix S5 Comparison of three algorithms (*Independent Swap*, *Richness*, *Frequency*) effect of null model on the results of considering MPD and CWM for every single traits (including leaf thickness (LT), Plant Height (PH), Leaf Shape (LS), Leaf Area (LA), Specific Leaf Area (SLA) and Leaf Dry Matter Content (LDMC) along with soil sodium (Na), potassium (K), Magnesium (Mg) and Nitrogen (N).

| **Soil elements** | **Functional traits** | ***Independent Swap*** | ***Richness*** | ***Frequency*** |
| --- | --- | --- | --- | --- |
| **S.E.S-MPD** | | | | |
| **Na** | LT | ***P-value*<0.05** | ***P-value*<0.05** | ***P-value*<0.05** |
|  | PH | *P-value*=0.51 | *P-value*=0.55 | *P-value*=0.98 |
|  | LS | *P-value*=0.23 | *P-value*=0.11 | *P-value*=0.12 |
|  | LA | *P-value*=0.62 | *P-value*=0.48 | *P-value*=0.29 |
|  | SLA | *P-value*=0.57 | *P-value*=1 | *P-value*=0.92 |
|  | LDMC | *P-value*=0.80 | *P-value*=0.27 | *P-value*=0.98 |
| **K** | LT | ***P-value*=0.07** | ***P-value*=0.08** | ***P-value*=0.09** |
|  | PH | ***P-value*=0.08** | ***P-value*=0.09** | ***P-value*=0.07** |
|  | LS | ***P-value*<0.01** | ***P-value*<0.05** | ***P-value*<0.05** |
|  | LA | *P-value*=0.74 | *P-value*=0.55 | *P-value*=0.38 |
|  | SLA | *P-value*=0.49 | *P-value*=0.76 | *P-value*=0.78 |
|  | LDMC | *P-value*=0.45 | *P-value*=0.45 | *P-value*=0.84 |
| **Mg** | LT | *P-value*=0.99 | *P-value*=0.91 | *P-value*=0.24 |
|  | PH | *P-value*=0.39 | *P-value*=1 | *P-value*=0.67 |
|  | LS | *P-value*=0.18 | *P-value*=0.15 | *P-value*=0.89 |
|  | LA | *P-value*=0.49 | *P-value*=0.71 | *P-value*=0.16 |
|  | SLA | *P-value*=0.53 | *P-value*=0.51 | *P-value*=0.49 |
|  | LDMC | *P-value*=0.46 | *P-value*=0.70 | *P-value*=0.23 |
| **N** | LT | *P-value*=0.68 | *P-value*=0.61 | *P-value*=0.98 |
|  | PH | *P-value*=0.13 | *P-value*=0.36 | *P-value*=0.25 |
|  | LS | *P-value*=0.33 | *P-value*=0.11 | *P-value*=0.20 |
|  | LA | ***P-value*=0.08** | ***P-value*=0.06** | ***P-value*=0.09** |
|  | SLA | *P-value*=0.73 | *P-value*=0.79 | *P-value*=0.87 |
|  | LDMC | *P-value*=0.45 | *P-value*=0.22 | *P-value*=0.65 |
| **S.E.S-CWM** | | | | |
| **Na** | LT | ***P-value*<0.01** | ***P-value*<0.01** | ***P-value*<0.01** |
|  | PH | *P-value*=0.92 | *P-value*=0.89 | *P-value*=0.91 |
|  | LS | *P-value*=0.51 | *P-value*=0.44 | *P-value*=0.44 |
|  | LA | ***P-value*<0.05** | ***P-value*<0.05** | ***P-value*<0.05** |
|  | SLA | ***P-value*=0.1** | ***P-value*=0.04** | ***P-value*=0.05** |
|  | LDMC | ***P-value*=0.1** | ***P-value*=0.07** | ***P-value*=0.07** |
| **K** | LT | *P-value*=0.24 | *P-value*=0.50 | *P-value*=0.47 |
|  | PH | *P-value*=0.52 | *P-value*=0.87 | *P-value*=0.79 |
|  | LS | ***P-value*<0.01** | ***P-value*<0.01** | ***P-value*<0.01** |
|  | LA | ***P-value*<0.05** | ***P-value*<0.05** | ***P-value*<0.01** |
|  | SLA | ***P-value*<0.05** | ***P-value*<0.05** | ***P-value*<0.05** |
|  | LDMC | *P-value*=0.47 | *P-value*=0.46 | *P-value*=0.45 |
| **Mg** | LT | ***P-value*<0.01** | ***P-value*<0.01** | ***P-value*<0.01** |
|  | PH | ***P-value*<0.01** | ***P-value*<0.01** | ***P-value*<0.01** |
|  | LS | ***P-value*<0.001** | ***P-value*<0.001** | ***P-value*<0.01** |
|  | LA | *P-value*=0.61 | *P-value*=0.71 | *P-value*=0.69 |
|  | SLA | ***P-value*<0.001** | ***P-value*<0.05** | ***P-value*<0.001** |
|  | LDMC | *P-value*=0.11 | *P-value*=0.17 | *P-value*=0.19 |
| **N** | LT | *P-value*=0.91 | *P-value*=0.96 | *P-value*=0.94 |
|  | PH | *P-value*=0.36 | *P-value*=0.19 | *P-value*=0.21 |
|  | LS | *P-value*=0.29 | *P-value*=0.26 | *P-value*=0.25 |
|  | LA | ***P-value*<0.001** | ***P-value*<0.001** | ***P-value*<0.001** |
|  | SLA | ***P-value*=0.1** | ***P-value*=0.06** | ***P-value*=0.06** |
|  | LDMC | *P-value*=0.44 | *P-value*=0.46 | *P-value*=0.49 |

Supporting Information to the paper Matinzadeh, Z. Functional structure of plant communities along salinity gradients in Iranian salt marshes. *Plant-Environment Interactions*.

Appendix S6 Principal component analysis (PCA) showing the contribution of nine selected soil variables.


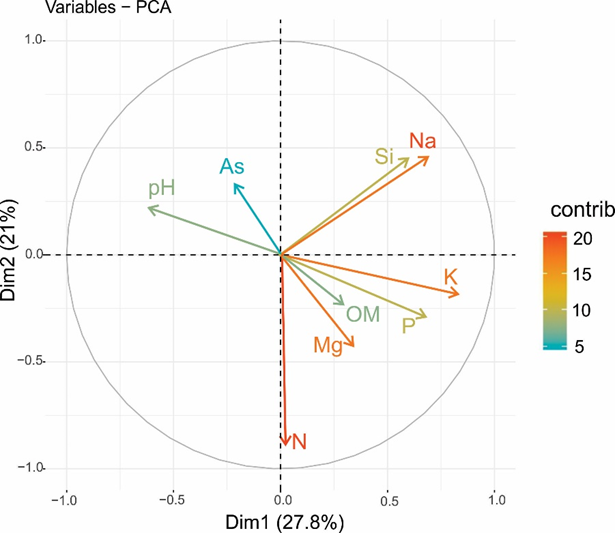

Supplement: Supplementary file 1 — DataS1 [file PEI3-3-10-s002.docx]
