# Supplementary material for: Chlamydomonas reinhardtii exhibits stress memory in the accumulation of triacylglycerols induced by nitrogen deprivation
Source: Plant Environ Interact. 2022 Mar 1;3(1):10–5. doi: 10.1002/pei3.10069 (PMC10168029; doi:10.1002/pei3.10069)
Supplement: Supplementary file 2 — FigS1 [file PEI3-3-10-s001.pdf]

a

Light setting	Measurement average
5LS	245 ± 11.9
4LS	144 ± 8.29
3LS	53 ± 2.94
2LS	35 ± 5.35
1LS	18 ± 2.83
	unit: umol m-2 s-1

b

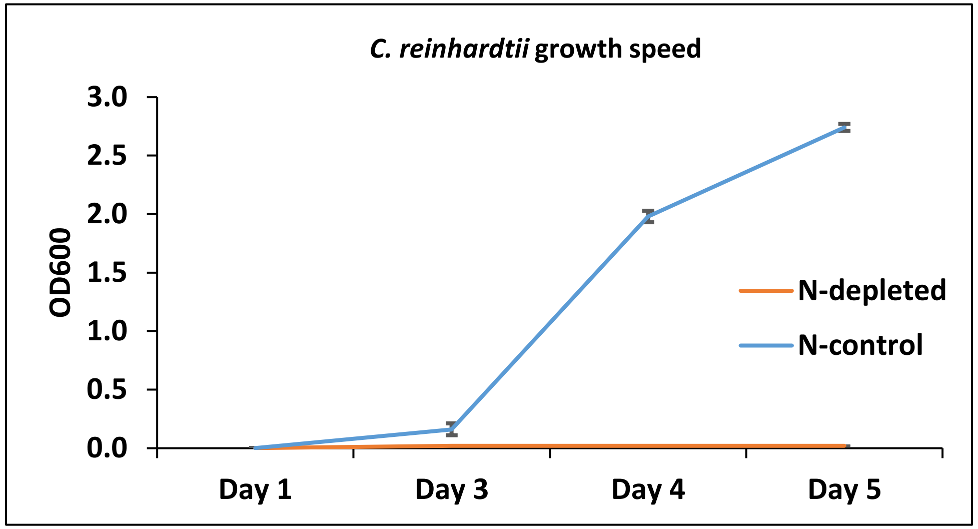

Fig.S1 Growth conditions' optimization. a. Light measurements in different settings of Sanyo 352-MLR-352-PE cabinets. Values indicated the average of 3 measurement in different positions on the growth chamber shelf. Plus-minus sign is followed by standard deviation from these measurements. Highlighted in red is the setting used for the further steps. **b.** Growth speed measurements in cells under nitrogen-control (in default TAP medium) or nitrogen-depleted conditions (TAP medium without nitrogen source). Error bars correspond to standard error from 2 biological replicates.
